# 3D printing titanium grid scaffold facilitates osteogenesis in mandibular segmental defects

**DOI:** 10.1038/s41536-023-00308-0

**Published:** 2023-07-24

**Authors:** Yongfeng Li, Huawei Liu, Chao Wang, Rongzeng Yan, Lei Xiang, Xiaodan Mu, Lingling Zheng, Changkui Liu, Min Hu

**Affiliations:** 1 Department of Stomatology, The First Medical Center of PLA General Hospital, Beijing, China; 2 Beijing Advanced Innovation Center for Biomedical Engineering, Beihang University, Beijing, 100083 China; 3 Nanchang University Fuzhou Medical College, Fuzhou, 344000 China; 4 Department of Oral and Maxillofacial Surgery, School of Stomatology, Xi’an Medical University, Xi’an, China

**Keywords:** Translational research, Preclinical research

## Abstract

Bone fusion of defect broken ends is the basis of the functional reconstruction of critical maxillofacial segmental bone defects. However, the currently available treatments do not easily achieve this goal. Therefore, this study aimed to fabricate 3D-printing titanium grid scaffolds, which possess sufficient pores and basic biomechanical strength to facilitate osteogenesis in order to accomplish bone fusion in mandibular segmental bone defects. The clinical trial was approved and supervised by the Medical Ethics Committee of the Chinese PLA General Hospital on March 28th, 2019 (Beijing, China. approval No. S2019–065–01), and registered in the clinical trials registry platform (registration number: ChiCTR2300072209). Titanium grid scaffolds were manufactured using selective laser melting and implanted in 20 beagle dogs with mandibular segmental defects. Half of the animals were treated with autologous bone chips and bone substances incorporated into the scaffolds; no additional filling was used for the rest of the animals. After 18 months of observation, radiological scanning and histological analysis in canine models revealed that the pores of regenerated bone were filled with titanium grid scaffolds and bone broken ends were integrated. Furthermore, three patients were treated with similar titanium grid scaffold implants in mandibular segmental defects; no mechanical complications were observed, and similar bone regeneration was observed in the reconstructed patients’ mandibles in the clinic. These results demonstrated that 3D-printing titanium grid scaffolds with sufficient pores and basic biomechanical strength could facilitate bone regeneration in large-segment mandibular bone defects.

## Introduction

Reconstruction of segmental maxillofacial bone defects after tumor, trauma, or infection remains a great challenge for clinicians, especially for critical segmental bone defects. Approximately 2.2 million patients suffer from bone defects related to orthopedics, neurosurgery, or dentistry^1^. Various strategies have been used to treat such clinical conditions, including distraction osteogenesis, allogeneic bone grafting, autologous bone grafting, and heterogeneous material implants. However, additional surgical trauma, insufficient donor resources, and various complications restrict the clinical application of the methods mentioned. Recent developments in the interdisciplinary field of tissue engineering have focused on restoring or maintaining tissue function using scaffolds, bioactive substances, and/or cells or tissues with regeneration potential^2^. Tissue engineering strategies have been utilized in the fields of plastics^3^, orthopedics^4^, and maxillofacial surgery^5^. Traditional tissue engineering processes for tissue implantation rely on ex vivo scaffolds combined with cells and biomolecules^2^. In situ tissue engineering, another approach for damaged tissue regeneration, regenerates tissue with the volume space of its intended functional site, leveraging the innate regenerative potential of the body. Compared with traditional ex vivo tissue engineering, seed stem cell harvesting and the establishment of complex cell culture conditions can be eliminated during in situ tissue engineering. Thus, in situ approaches can be more favorably translated into a clinical context than ex vivo tissue engineering ones^6^, especially in the field of bone grafting for orthopedics^7^ or maxillofacial applications^8,9^.

**Fig. 1:**
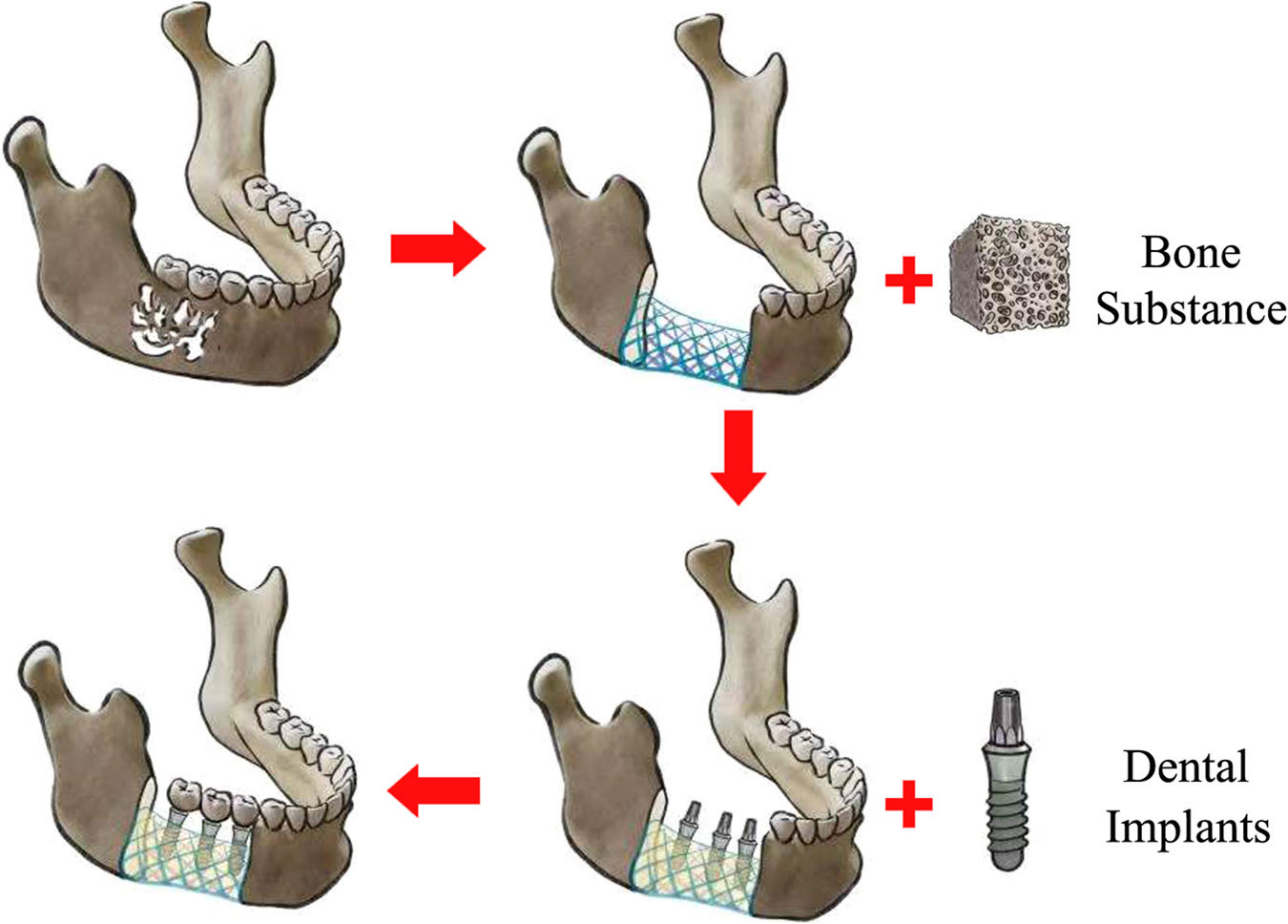
The design concept of this study. Critical segmental bone defect reconstructed by a reinforced concrete structure consisting of 3D printing grid scaffold + bone substances, dental implant insertion surgery can be performed after regenerated new bone suffused porosity of scaffold, and finally realized the purpose of occlusal function restoration.

Based on our previous findings^10–12^, we attempted to fabricate an in situ tissue-engineered construct at the defect region using a 3D scaffold, with dual effects of favorable mechanical properties to resist fatigue and create sufficient interspace for vascularization^13^ (Fig. 1) . The development of 3D printing has enabled the fabrication of scaffolds with sufficient porosity^14,15^. Multiple researchers have analyzed the biomechanical and biocompatible properties of 3D-printing scaffolds via finite element analysis (FEA), biomechanical tests, and in vitro experiments^16–20^. After an 83-year-old female patient underwent a specific titanium jaw prosthesis implantation fabricated with the selective laser melting (SLM) technique in 2011, a series of clinical studies have attempted to reconstruct mandibular defect using 3D printing prothesis^17–21^. Moreover, in vivo, experiments and clinical trials from orthopedic studies have reported bone ingrowth into the pores of 3D-printing porous structures^22^ or mesh scaffolds^23^. However, continuous bone fusion from broken bone ends was not investigated in these studies, which is crucial for subsequent dental implant placement in the field of dentistry. Therefore, we attempted to realize continuous bone fusion from bone broken ends via 3D-printing Ti-grid scaffolds in animal experiments and clinical trials.

## Results

### Scaffold design and property

**Fig. 2:**
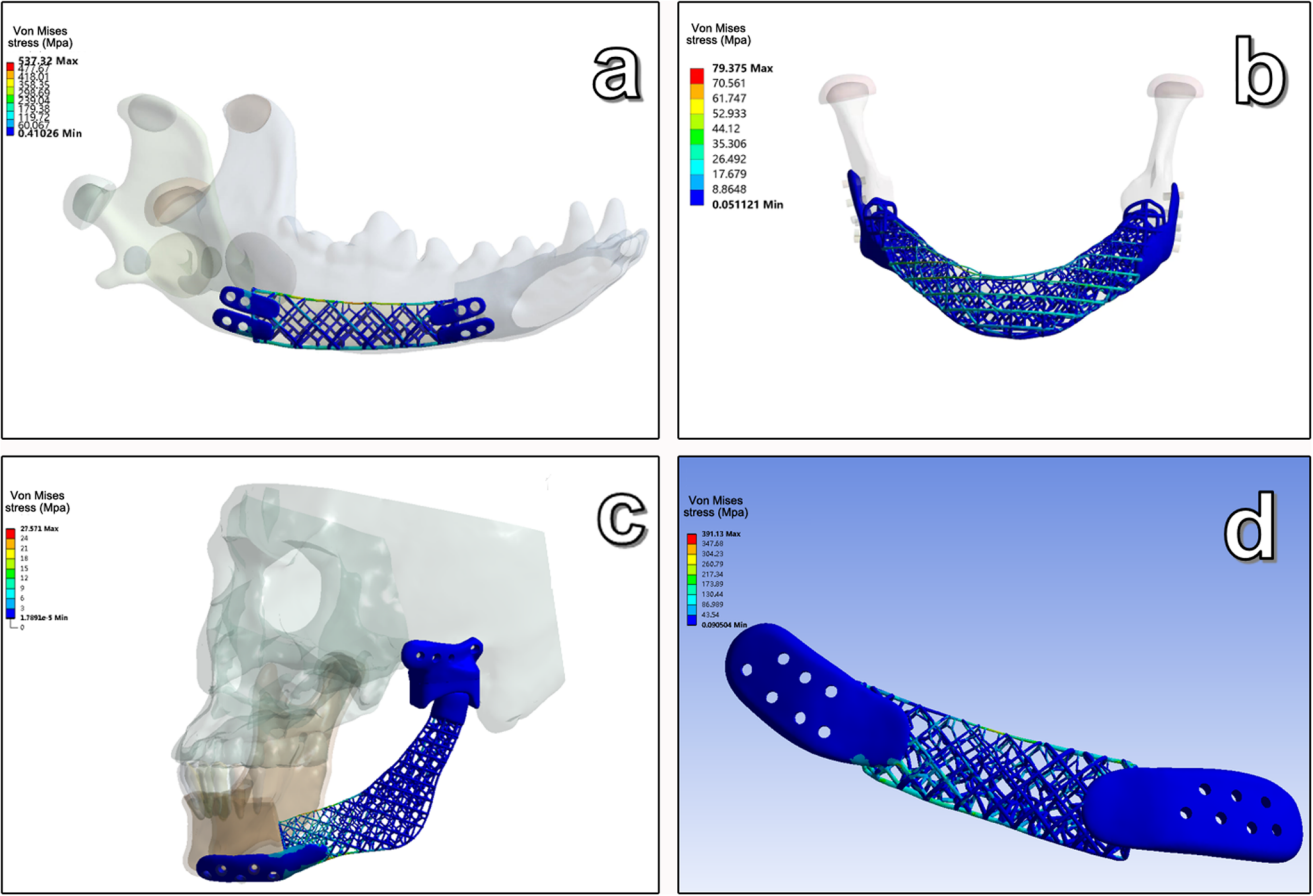
Finite element analysis of the scaffolds. **a** Von Mises stresses of scaffolds in animal experiments. **b** Von Mises stresses of scaffolds in case 1. **c** Von Mises stresses of scaffolds in case 2. **d** Von Mises stresses of scaffolds in case 3.

The porosity of the Ti-grid scaffolds for the animal experiment in Group 1 and Group 2 was 92.76 (2.77)% and 92.17 (2.39)%, respectively. No differences were found between the groups. The scaffold had a 3D internal gradient structure with pore sizes varying from 3 mm at the inferior border to 5 mm at the superior border of the scaffold. The diameter of the trabeculae varied from 0.3 at the superior border to 0.5 mm at the inferior border of the scaffold. The results from the FEA calculation showed that the maximum von Mises stress in the scaffold for the animal experiment was 537.32 MPa, which was concentrated in the superior region of the scaffold (Fig. 2a). Moreover, the maximum von Mises stress values in the scaffold for cases 1–3 were 79.375, 27.571, and 391.13 MPa, respectively (Fig. 2b–d). The von Mises stress values of the scaffolds for animal experiments and clinical trials were all lower than the yield strength of Titanium (897 MPa). These results indicated that the mechanical properties of the optimized 3D grid scaffolds could meet basic biomechanical demands.

### Animal experiments

In an animal experiment, we investigated whether bone fusion was achieved from bone-broken ends using radiological scanning and histological analysis. Of the 20 animals, three beagle dogs (including one in Group 2 and two in Group 1) were excluded from this study because of anesthesia or scaffold fracture due to unanticipated overloaded biting (i.e., metal cage).

### In vivo observation

Intraoral scaffold exposure was observed in three animals in Group 1 and two animals in Group 2. The data obtained from computed tomography (CT) showed that there was no apparent scaffold fragment or displacement and some hyperintensity characteristics inside the scaffold in Group 1, indicating the formation of mineralized bone islands. In contrast, a continuous hyperintensity was observed in the scaffold. Continuous stretches of mineralized tissue were observed in both groups 18 months after scaffold implantation. However, the volume of regenerated osseous tissue in Group 2 was greater than that in Group 1. The area of osteogenesis in Group 1 was mainly concentrated at the superior side of the scaffold (Supplementary Fig. 2). The results from positron emission tomography (PET)/CT revealed continued metabolic activity in the scaffold of the two groups 18 months after implantation, indicating the persistent nature of osteogenic bones and sufficient vascular supply (Supplementary Fig. 3).

### Micro-CT scanning

Animals were sacrificed for micro-CT scanning, and the bone architecture was analyzed 18 months after scaffold implantation. This analysis showed continuous trabecular structure growth in the scaffold pores. In general, most of the scaffolds in Group 2 were filled with regenerated new bone, whereas the osteogenic area in Group 1 was smaller than that in Group 2 (Supplementary Fig. 4). Moreover, the ratio of mineral bone tissue volume to that of total tissue in Group 2 29.31 (9.74)% was significantly higher than that in Group 1 [22.2 (7.44)%] (*p* < 0.05). The density of the mineral bone in the distal area was also higher than that in the middle and mesial areas on coronal images and superior areas with higher density than inferior areas on sagittal images.

### Histological analysis

A histomorphological evaluation was performed on a thin section of each sample in the coronal plane through the center of the Ti-grid scaffolds in Group 1 and the sagittal plane through the center of the scaffolds in Group 2. The resulting fluorescence micrographs showed that the mineral tissue (labeled with calcein and tetracycline) was deposited along the new bone surfaces, as green and yellow fluorescence, respectively (Supplementary Fig. 5). The rate of new bone mineralization, defined as the bone mineralization apposition ratio (MAR, µm/day), was determined based on the distance between the two fluorescent markers. The MAR was 2.65 (0.55) µm/day and 2.12 (0.62) µm/day in Group 1 and Group 2, respectively, without any significant differences.

Following fluorescence observation, the regenerated new bone in the scaffold was examined in undecalcified sections stained with methylene blue/acid fuchsin, which distinguishes calcified bone from other tissues by giving it a bright pink color. The Ti-grid scaffold was stained black, and the bone substitute material was stained brown on the slides. Images from Group 1 showed an osteogenic area concentrated at the superior and lingual sites, whereas the other areas of the scaffold were filled with non-mineralized fibrous and adipose tissues. Moreover, analysis at high magnification revealed typical onion-like Haversian systems, mainly located in the peripheral regions of the osteogenic area. In Group 2, the pores of the scaffolds were almost filled with regenerated new bone, only 1–3 mm at the superior and inferior margins of the scaffold, and the mineralized tissue was arranged in band-like patterns from distal to mesial areas. The newly formed bone tissue was aligned with a normal trabecular structure, and the volume of bone tissue in the distal area was higher than that in the mesial area. A distinct fusion line was detected at the margin of the newly formed bone tissue and basal bone, with an inferior-to-superior orientation. Additionally, a vasculoid structure was observed in the intraosseous and bone marrow cavities. At high magnification, a brown-stained, scattered bone substitute was distributed in the osteogenic area, and pink-stained mineralized tissue was also deposited at the margin or center of the bone substitute. For quantitative analysis, the ratios of bone volume to total volume (BV/TV) in Group 1 and Group 2 were 22.02 (6.52)% and 27.36 (9.4)%, respectively, the values of %Tb.Ar in Group 1 and Group 2 were 54.21 (11.86)% and 44.19 (9.24)%, respectively, with significant differences between the groups.

**Fig. 3:**
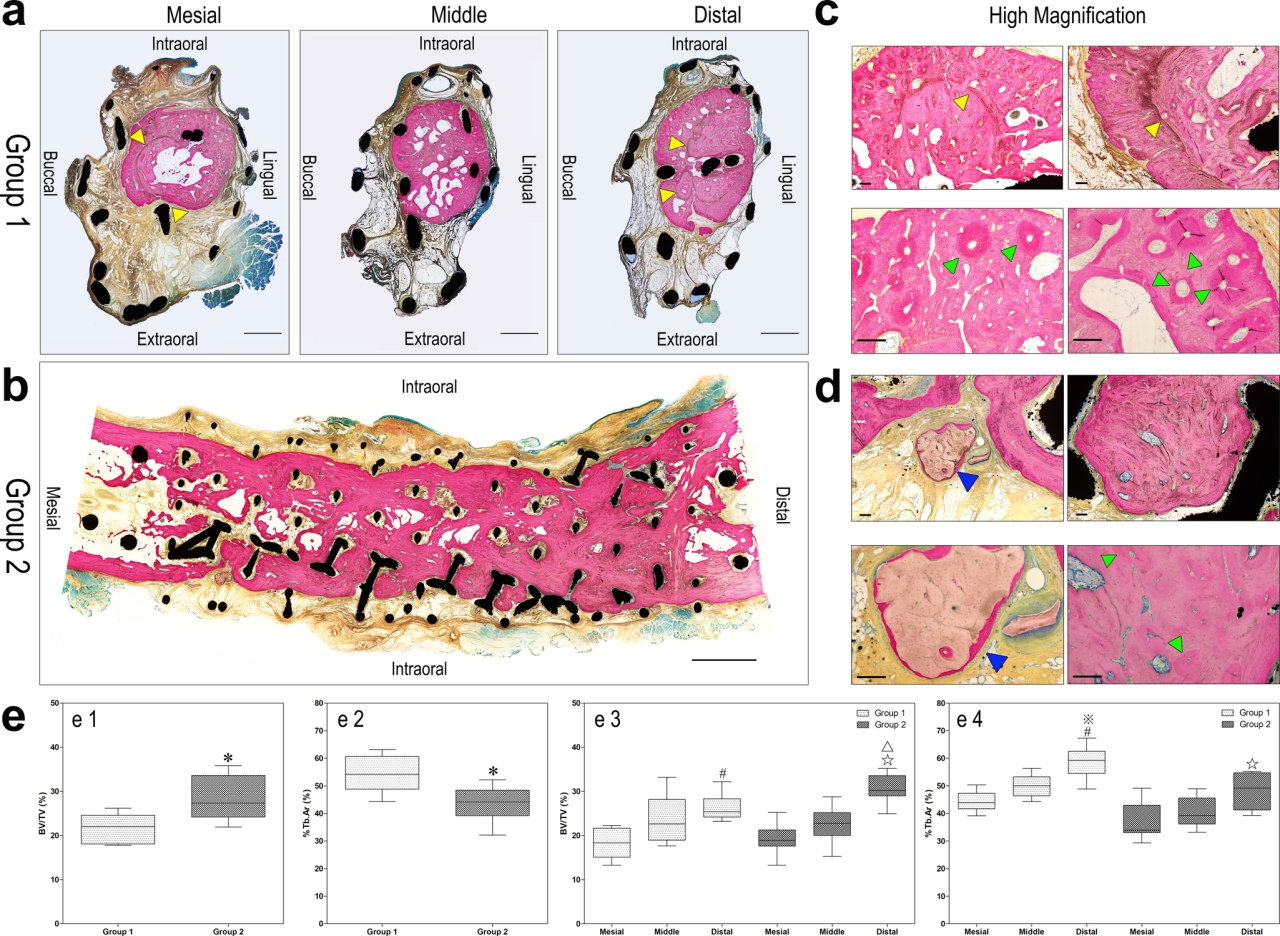
Histological analysis to investigate new bone formation stained with methylene blue/acid fuchsin. The calcified bone was stained a bright pink color, the Ti-mesh scaffold was stained black, and the bone substitute was stained brown in the slides (blue triangles), Havers’ systems were observed at high magnification (green triangles). **a** Histological section observation of Group 1 (low magnification). Scale bar, 2 mm. **b** Histological section observation of Group 2 (low magnification). Scale bar, 5 mm. **c** Histological section observation of Group 1 (high magnification). Scale bar, 200 µm. **d** Histological section observation of Group 2 (high magnification). Scale bar, 200 µm. **e** 1. Comparison of BV/TV in groups 1 and 2. **e** 2. Comparison of %Tb.Ar in groups 1 and 2. **e** 3. Comparison of BV/TV at the mesial, middle, and distal areas in groups 1 and 2. **e** 4. Comparison of %Tb.Ar at the mesial, middle, and distal area in groups 1 and 2 (*P < 0.05 compared to Group 1; #*P* < 0.05 compared with mesial area in Group 1; ※*P* < 0.05 compared with middle area in Group 1; ☆*P* < 0.05 compared with mesial area in Group 2; Δ*P* < 0.05 compared with middle area in Group 2).

For the osteogenic area, the BV/TV values at the distal, middle, and mesial areas in Group 1 were 25.4 (4.13)%, 22.65 (9.22)%, and 18.34 (6.54)%, respectively. A significant difference was observed between the distal and mesial areas. The value of %Tb.Ar at the mesial, middle, and distal areas were 43.90 (5.55)%, 50.00 (6.91)%, 59.25 (8.00)%, respectively, and the %Tb.Ar of the distal area was significantly higher than that of the middle and mesial areas. For Group 2, the BV/TV values at the distal, middle, and mesial areas in Group 1 were 30.22 (4.59)%, 22.758 (5.15)%, and 18.93 (3.66)%, respectively, which were significantly higher than those in the other two areas, although the BV/TV value was higher in the middle area than in the mesial area, with no significant difference. The %Tb.Ar values at the mesial, middle, and distal areas were 33.97 (9.91)%, 39.28 (9.40)%, 49.19 (13.46)%, respectively, and a significant difference was only observed between the mesial and distal areas (Fig. 3).

### Clinical trials

Of the three patients involved in this study, the operation proceeded successfully, and individualized scaffold placement coincided with the position of neoplasm resection. The postoperative contour was satisfactory in all three patients, with no evidence of common complications, such as scaffold fracture, loosening, exposure, and facial paralysis related to facial nerve injuries.

**Fig. 4:**
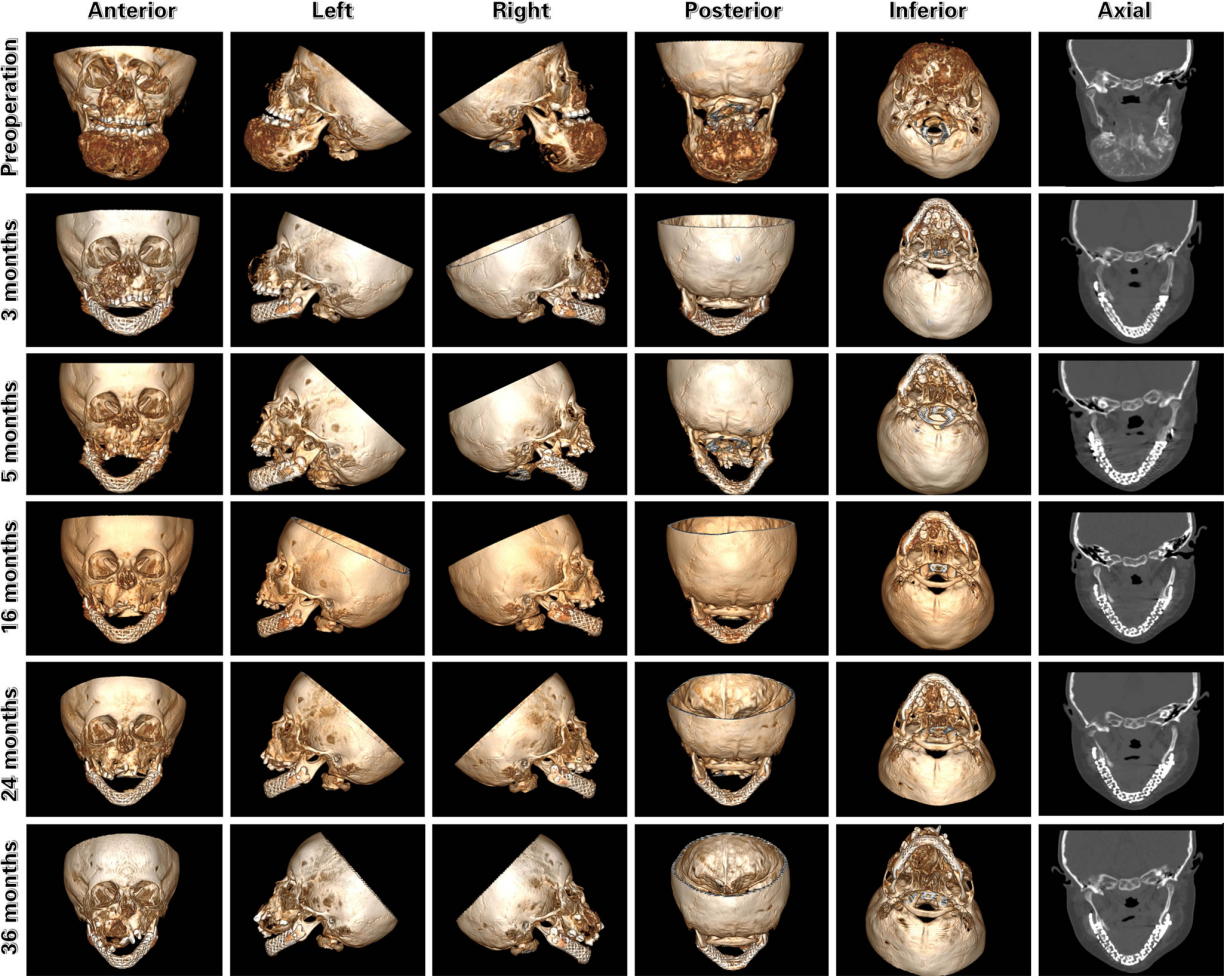
Spiral CT scanning following up on case 1. The radiological images indicated that no displacement, loosen or fracture of scaffold occurred, and mineralization of tissues was detected in the scaffold concentrated at the chin and the distal area of the scaffold.

In Case 1, the patient was followed up for three years (Fig. 4); Two weeks after the Ti-grid scaffold placement, the patient enjoyed her first normal meal and favorable sleeping in nearly 1 year (as described by the parents of the child). Another positive result was an increase in the weight of the child by 5 kg at the 1-month follow-up. Unfortunately, the patient underwent two modified operations for the same myxofibroma on the maxilla. Regular CT scans at 3, 16, 24, and 36 months indicated that the scaffold was integrated with the lateral mandibular ramus. Meanwhile, despite the interference of metallic artifacts, mineralization of tissues was detected in the scaffold concentrated at the chin and the distal area of the scaffold, with the Hounsfield unit (HU) value of mineralization tissue being approximately 450, which is comparable to the bone of the maxilla and atlas (Fig. 4). The emission CT (ECT) images showed significant radioisotope concentrations in the middle and left regions of the scaffold, which suggested the development of vascularized bone tissue (Supplementary Fig. 8). The high radioisotope concentration in the maxillary region indicated a neoplasm, that was previously demonstrated to be the same myxofibroma.

In Case 2, the patient was followed up for only 6 months. The symptoms of the mandible with left deviation during mouth opening improved significantly, and the occlusal relationship was normal (Supplementary Fig. 13). The temporomandibular joint was steady during functional movement, which included opening, closing, and lateral movements of the mandible with no deviation or blocked or dislocated symptoms. Furthermore, the CT images showed that the junction of the scaffold and residual bone was in tight contact, and the 3D relationship between the condyle and temporomandibular fossa was normal (Supplementary Fig. 14).

In Case 3, there was no tumor recurrence occurred at the 6-month follow-up. The contour was satisfactory for the patient, and no intraoral or extraoral scaffold exposure was observed. The centerlines of the lower and upper incisors were aligned, and the occlusal relationship was normal (Supplementary Fig. 15). Mineralized tissue was detected in the honeycomb of the scaffold after 6 months, and the HU value was approximately 450, which is comparable to the cancellous bone of the contralateral mandible (Supplementary Fig. 16).

## Discussion

From the results of animal experiments, favorable osteogenesis occurred in the pores of the Ti grid scaffolds, and further consecutive bone bridges performed bone fusion from bone broken ends, which ensured subsequent dental implant placement for functional maxillofacial reconstruction. This phenomenon demonstrated that 3D printing Ti grid scaffolds with sufficient space could act as an available scaffold for tissue engineering to facilitate osteogenesis. The concept of “tissue engineering” was introduced by Green in the early 1970s, who performed several experiments to generate cartilage from chondrocytes seeded in spicules^2,24^. Since then, this technique has gained considerable attention in the field of tissue defect reconstruction, including bone, cartilage, and vascular reconstruction. Bone tissue engineering is one of the earliest and most popular applications of this technique. Although bone tissue engineering has progressed for nearly half a century, few associated technologies have been translated into routine clinical practice, especially large-segment bone defect reconstruction. The three basic elements of engineering are seed cells, scaffolds, and growth factors. In ex vivo tissue engineering, scaffolds are combined with seed cells and growth factors outside the body to obtain cell-laden tissue constructs for implantation, and nutrient supply and metabolic waste clearance for tissue fabrication are realized under ideal circumstances. Comparable functions were achieved by surrounding vascular in situ tissue engineering. A review published in 2019 proposed that more in situ tissue engineering is available for clinical use than ex vivo systems, including bone tissue, while some tissues, such as cartilage, cardiac, and central nervous systems, fail to regenerate because of a limited supply of endogenous cells^6^. Therefore, bone tissue with a rich blood supply is a suitable choice for implementing in situ tissue engineering strategies in clinical applications.

In this study, three basic elements were involved in in situ tissue engineering: the 3D-printing Ti-grid was the scaffold, primary seed cells, and growth factors were recruited from the surrounding vasculature, and autologous bone fragments and allogeneic bone materials enhanced cell recruitment and provided exogenous growth factors.

The grid design ensured as much porosity as possible on the premise of maintaining the basic biomechanical strength of the mandibular function. The scaffolds used in the animal experiments and clinical trials were mechanically optimized. FEA showed that the maximum von Mises stress distribution of the scaffolds was lower than the yield strength of Ti in both animal and clinical applications. The loaded strength is highest at the early stage of scaffold placement; a significant decrease in the stress distribution occurs owing to bone tissue or other fibrous tissue ingrowth into the scaffold^25,26^. Based on mechanical optimization, the pore size of the scaffold was designed to be 3–5 mm, and the trabecula was designed to be 0.2–0.5 mm to reduce the amount of Ti used. This design facilitates the exchange of nutrients and functional cells. Meanwhile, pore sizes can determine how stem cells differentiate; as described in previous studies^27,28^: pore sizes >200 µm are more suitable for the formation of unmineralized and fully mineralized bone tissues. For example, bone formation requires a minimum pore size of 100–150 µm, whereas *t* vascularization requires pores >300 µm. Generally, a pore size of 50–1000 µm is recommended for cell growth and full recovery.

The challenge of Ti-grid scaffold fabrication is that the porosity exceeds 90%. Fortunately, the 3D printing technique facilitated the process with the available metal 3D printing strategies, including SLM and electron beam melting (EBM). Previous studies have verified the effectiveness and safety of Ti implants in maxillofacial bone defect reconstruction fabricated with SLM or EBM after a long-term follow-up^17,29^. SLM is an additive manufacturing technique in which objects are fabricated by melting and solidifying successive layers of powdered materials, and 3D objects are fabricated by repeating these steps for every layer. EBM is a powder-bed fusion technique that involves dividing each slice into two regions and fabricating the required parts within these boundaries^11,30–32^. Compared to SLM, the scaffold fabricated by EBM possesses favorable mechanical properties for higher energy but with a lower accuracy compared to SLM^16,33–35^. In this study, we applied the SLM technique to scaffold fabrication because of the high demand for accuracy, with the mandible not being the bearing area.

Maxillofacial bones have been targets of 3D printing techniques in bone defect reconstruction primarily due to the fact they have higher patient-specific requirements than other regions. 3D printing combined with computer-aided design can accomplish complex and highly personalized structure fabrication. 3D printing for mandibular prostheses has been reported by clinicians since 2011. To accomplish the ultimate aim of mandibular reconstruction—occlusal function restoration, a design with a pre-mount dental implant and a 3D printing mandibular prosthesis was proposed. Lee et al. fabricated a porous mandibular prosthesis combined with pre-mount dental implants that penetrated gingival healing; however, the abutment was curetted due to infection around the abutment^20^. Kim et al. installed conventional dental implants in the hole of a mandibular prosthesis before prosthesis implantation, where the dental implants were completely submerged inside the oral mucosa, and the crown and abutment were placed after soft tissue healing. Unfortunately, unfavorable mucosal healing disturbed the anticipated restoration plan^18^. Jo et al. reconstructed a mandibular discontinuous defect with a porous customized Ti implant, which was fabricated using EBM technology, although a small amount of new bone migration was observed in the porous area^36^. Although there were some limitations restricting final occlusal function restoration, especially unfavorable soft tissue healing around implants, some details from these studies are important to note: submerging healing for the success of the dental implant was important, and the design of the porous structure was beneficial for bone tissue migration. Moreover, previous studies found that the soft tissue-to-bone interface could resist infection better than the soft tissue-to-metal interface^37–40^. Therefore, we expected to overcome the associated limitations by promoting sufficient and high-quality osteogenesis around the prosthesis, which can protect soft tissue from infection and exposure, and subsequently insert dental implants into the regenerated bone for ultimate occlusal function restoration.

Fortunately, results from in vivo tests, such as spiral CT, ECT, and PET/CT, indicated that the HU values of the tissues in the scaffold were comparable to those of other maxillofacial bones in Cases 1 and 3. The PET/CT images from the animal experiment displayed remarkable radioactive concentrations in the surgical regions in both Groups 1 and 2, which demonstrated the presence of vascularized active bone metabolism in the pores of the scaffold. A similar phenomenon was observed in the middle and left regions of the scaffold in Case 1. Subsequent histological analysis confirmed the mineralization of the bone tissue that formed inside the scaffold. Further investigation revealed that the amount of osteogenic bone decreased from the distal to the mesial area, and the bone tissue was concentrated in the central, lingual, and intraoral regions of the scaffold instead of the peripheral buccal and extraoral regions. This phenomenon indicates that nutrition for osteogenesis is mainly derived from the central blood supply. The blood supply to the mandible mainly includes the central inferior alveolar neurovascular bundle and peripheral intraperiosteal vessels. In this study, the central inferior alveolar neurovascular bundle was reserved at the lingual side of the mandible in animal experiments to provide an adequate central blood supply from the distal to the mesial areas. Peripheral intraperitoneal blood supply plays a subordinate role in segmental mandibular bone osteogenesis. Moreover, the discrepancy in osteogenesis between the intraoral and extraoral regions could be attributed to the para-oral incision from the skin. In this study, the teeth were extracted in advance, and the oral mucosa was allowed to heal entirely before resection surgery and reconstructive therapy. Compared to the extraoral region, complete intraperitoneal injection in the gingiva after tooth extraction provided superior blood supply reconstruction. The para-oral incision is the traditional choice for segmental mandibular resection and reconstruction^41,42^. A similar surgical strategy and active osteogenesis area were observed in Case 1. The potential mechanism is the use of the body as a bioreactor based on the arteriovenous loop or bundle^43–45^. The strategies applied in our study were all favorable for clinical use, including vascular reservation, surgical incision choice, and surgical strategy application.

Although various bone grafting strategies have been used to manage segment bone defect reconstruction^46–48^, the potential osteogenic mechanism remains unclear. Further investigation of histological images may provide some clues. Group 1 showed abundant Haversian systems arranged in the peripheral region of the newly formed bone and some block-like bone tissues with obvious boundaries inside the bone tissue (Fig. 3). The Haversian system was also arranged in the peripheral region of the inner block-like bone tissues. Mature bone consists of peripheral cortical bone and central trabecular bone, with regularly arranged Haversian cortical bone systems indicating that one osteogenic cycle is complete. Therefore, we hypothesized that the inner block-like bone tissue is the primary osteogenic core. The subsequent osteogenic procedure developed around the primary osteogenic core, ultimately forming the final bone structure. The primary osteogenic core determines the final osteogenic region, but primary osteogenic core formation seems spontaneous and unpredictable. The range of regenerated bone in the scaffold in Group 2 was larger than that in Group 1, which may be due to the widespread multiple osteogenic cores derived from autologous bone chips combined with bone substitutes that filled the scaffolds. The phenomenon of the lower density of the bone tissue in Group 2 than in Group 1 suggests that spontaneous osteogenesis without bone substitutes would favor the formation of an ideal bone microstructure. Meanwhile, the quantitative analysis from PET/CT and double fluorescence also reflected similarly higher bone metabolism and mineralization in Group 1. However, there were no significant differences between the two groups.

The cases involved in this study consisted of three age groups: infants with rapid growth and development, middle-aged patients with stable metabolism, and older patients with decreased metabolism. Scaffold implantation in subjects from the three age groups was accomplished with favorable contour reconstruction. Moreover, from the aspect of the bone defect region, the scaffolds reconstructed three types of mandibular segmental defects: the bilateral mandible body, the unilateral partial mandible body with the joint, and the unilateral partial mandible body. The aforementioned segmental bone defect types also represent different mechanical and osteogenic demands. After mechanobiological optimization, the scaffolds were found to be suitable for normal physiological movement with lesser amounts of titanium and promoted osteogenesis simultaneously in Cases 1 and 3. Case 3 proved to be the most promising for widespread clinical application and was classified as prosthesis replacement after mechanical complications of the original prosthesis. This type of case has the following advantages: First, the mechanical difficulty of the original prosthesis, including fracture, loosening, or fatigue, reflects the unfavorable mechanical design of the original prosthesis. Moreover, mechanobiological optimized prostheses would decrease the risk of mechanical complications. In addition to Cases 1 and 2, the teeth were extracted in the previous surgery, and the oral mucosa was allowed to heal entirely before reconstructive therapy. This surgical strategy protected the intraoral mucosal blood supply for tissue healing and osteogenesis despite the decreased radiotherapy-induced reduced blood supply.

Despite the limited sample size and evaluation methods restricting further comprehension of the osteogenic mechanism in segment bone defect reconstruction, available data from animal experiments and clinical trials have shown that this technique can realize favorable osteogenesis and further bony structure continuity reconstruction.

## Methods

### Study design

This study aimed to fabricate 3D-printed titanium grid scaffolds and maintain the basic biomechanical strength of the mandibular function. The porosity of the scaffold should be increased to the maximum possible extent. We also evaluated the effects of bone regeneration on mandibular segmental defects in animal experiments and clinical trials. In this study, we fabricated similar mechanical titanium grid scaffolds using the FE technique to ensure resistance to typical mechanical failure. We established 4-cm segmental mandibular defects in 20 beagle dogs, which were divided into two experimental groups to evaluate their osteogenic effects in vivo. Three patients with segmental mandibular defects were treated with 3D-printing titanium grid scaffold implantation surgery to investigate their clinical impact.

### Ethics approval statement

**Fig. 5:**
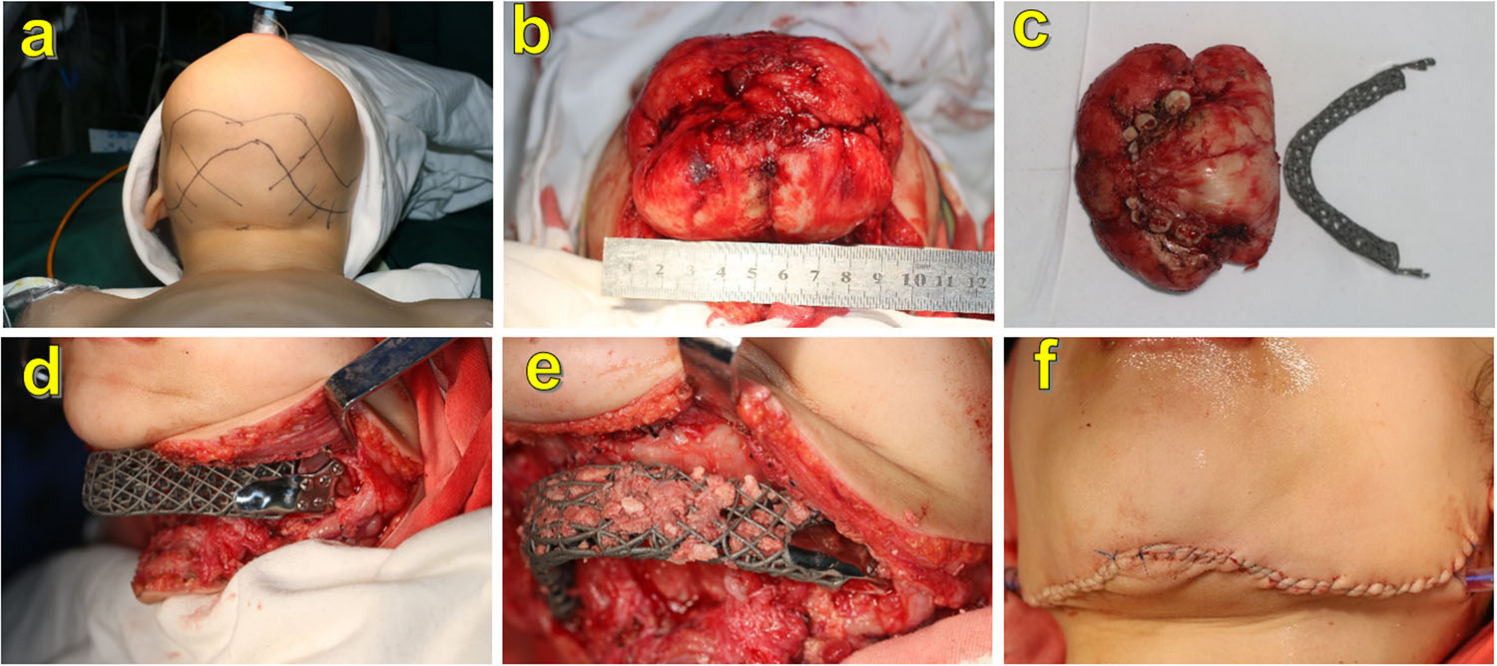
The procedures of scaffold implantation in case 1. Surgical incision design. **a** Exposure to neoplasm. **b** Neoplasm excision. **c** Scaffold implantation and fixation. **d**, **e** Autogenous bone fragment filled into the pore of the scaffold. **f** Suture.

**Fig. 6:**
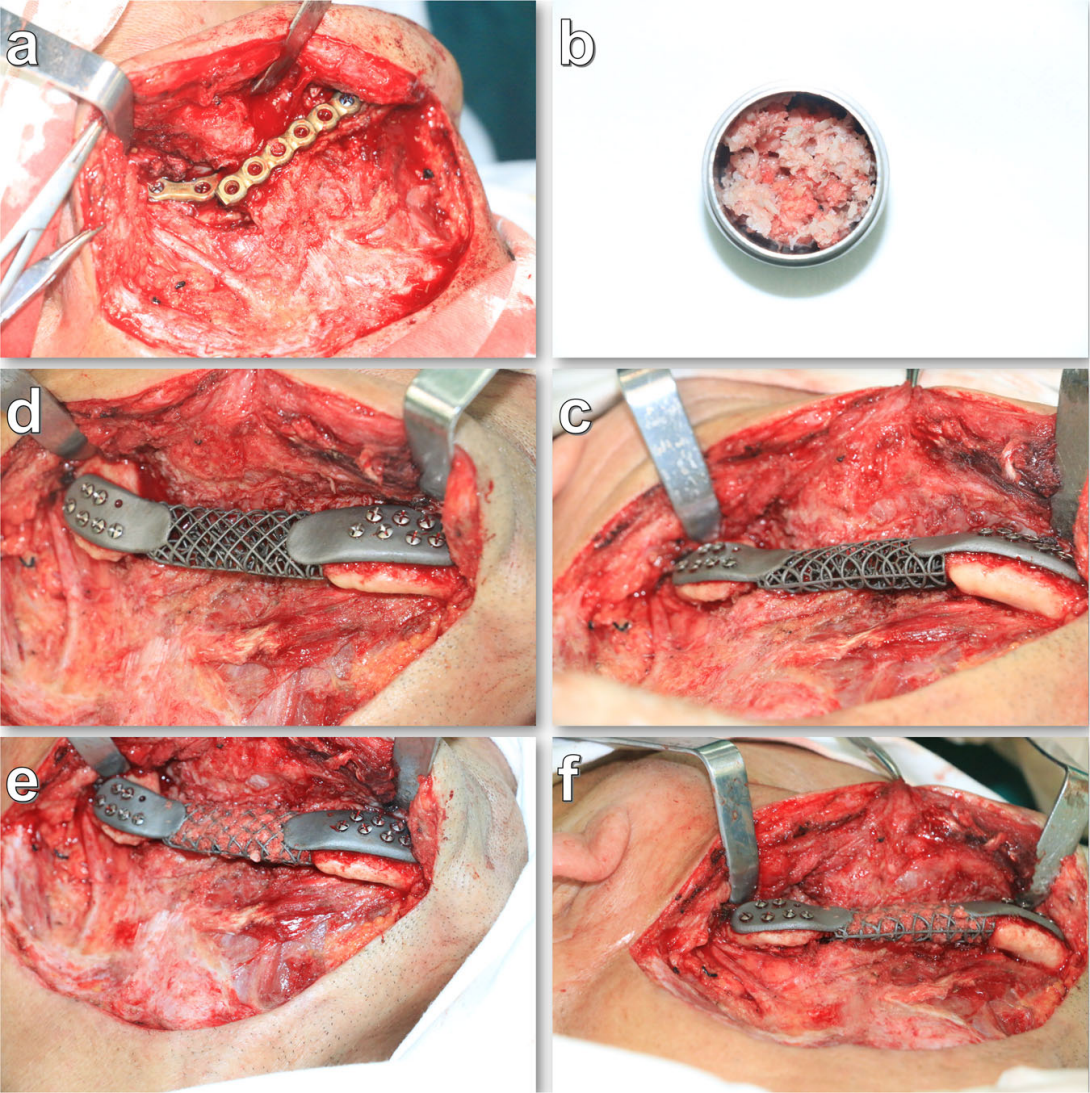
The procedures of scaffold implantation in case 3. **a** Exposure of fractured Ti plate. **b** Autogenous bone fragment preparation by the bone mill. **c**, **d** Scaffold implantation and fixation. **e**, **f** Autogenous bone fragments filled into the pore of the scaffold.

Animal experiment procedures were conducted according to the guidelines of the Ethical Review of Laboratory Animal Welfare (GB/T358922018) and approved and supervised by the Institutional Animal Care and Use Committee of the Chinese PLA General Hospital (Beijing, China. approval no. 2017-D13–15). The clinical trial was approved and supervised by the Medical Ethics Committee of the Chinese PLA General Hospital on March 28th, 2019 (Beijing, China. approval No. S2019–065–01), and registrated in the clinical trials registry platform (registration number: ChiCTR2300072209). Written informed consent was obtained from all patients or their guardians, and the authors affirmed that human research participants provided informed consent for publication of the images in Figs. 5 and 6, as well as Supplementary Figs. 9, 12, 13, and 15.

### Scaffold-optimized design and fabrication

Based on a previous study^10^, a diamond unit cell was used to construct a trabecular-like structure through random distribution, and 3D models of these structures were developed using 3-Matic software (Materialize, Leuven, Belgium). A pore diameter, ranging from 5 to 8 mm, was chosen for easy filling with autogenous bone particles or biomaterials, and the struct diameters were 0.3–0.5 mm.

To study the biomechanical properties of the scaffolds under occlusal conditions, three load cases were simulated: vertical loading with 100 N (loading I), inclined loading with a 30° angle of 100 N (loading II), and vertical loading with 200 N (loading III). According to the von Mises yield criterion, the mechanical properties of the different scaffolds were evaluated by comparing the maximum von Mises stress with their yield strength (897 MPa)^49^. Therefore, maximum safety to avoid mechanical failure was used as a criterion to assess the configuration.

3D Ti-grid scaffolds were fabricated using SLM (Concept Laser, Concept Laser GmbH, Germany). A scaffold was printed from the inferior border to the superior edge of the mandible. Before the preclinical in vivo model, the scaffold underwent a series of post-processing procedures, including removal of the support parts, dedusting, acid etching, thermal treatment, and polishing and sterilization (Supplementary Fig. 1, Supplementary Fig. 7, Supplementary Fig. 11, and Fig. 6).

### Animal experiments

Twenty adult beagle dogs (weight: 12 ± 3.4 kg; age: >2 years) were used in this study. Before scaffold implantation, unilateral molars and premolars of beagle dogs were extracted under general anesthesia with pentobarbital (3%, 30 mg/kg, Merck Drugs & Biotechnology, Germany) intravenous injection, and endotracheal intubation with 2% sevoflurane anesthesia, analgesia was performed by intramuscular injection with Bucinazine hydrochloride (4 mg/kg) for 3 days post-operation. Moreover, antibiotics (ampicillin, 12.5 mg/kg, China) were administered for five days to avoid infection after surgery. Three months after tooth extraction, spiral CT (Philips Brilliance iCT, Philips, Netherlands) of the mandibular region of the beagle dogs was performed under general anesthesia. According to the above procedure, the DICOM datum was used for scaffold design and optimization.

**Fig. 7:**
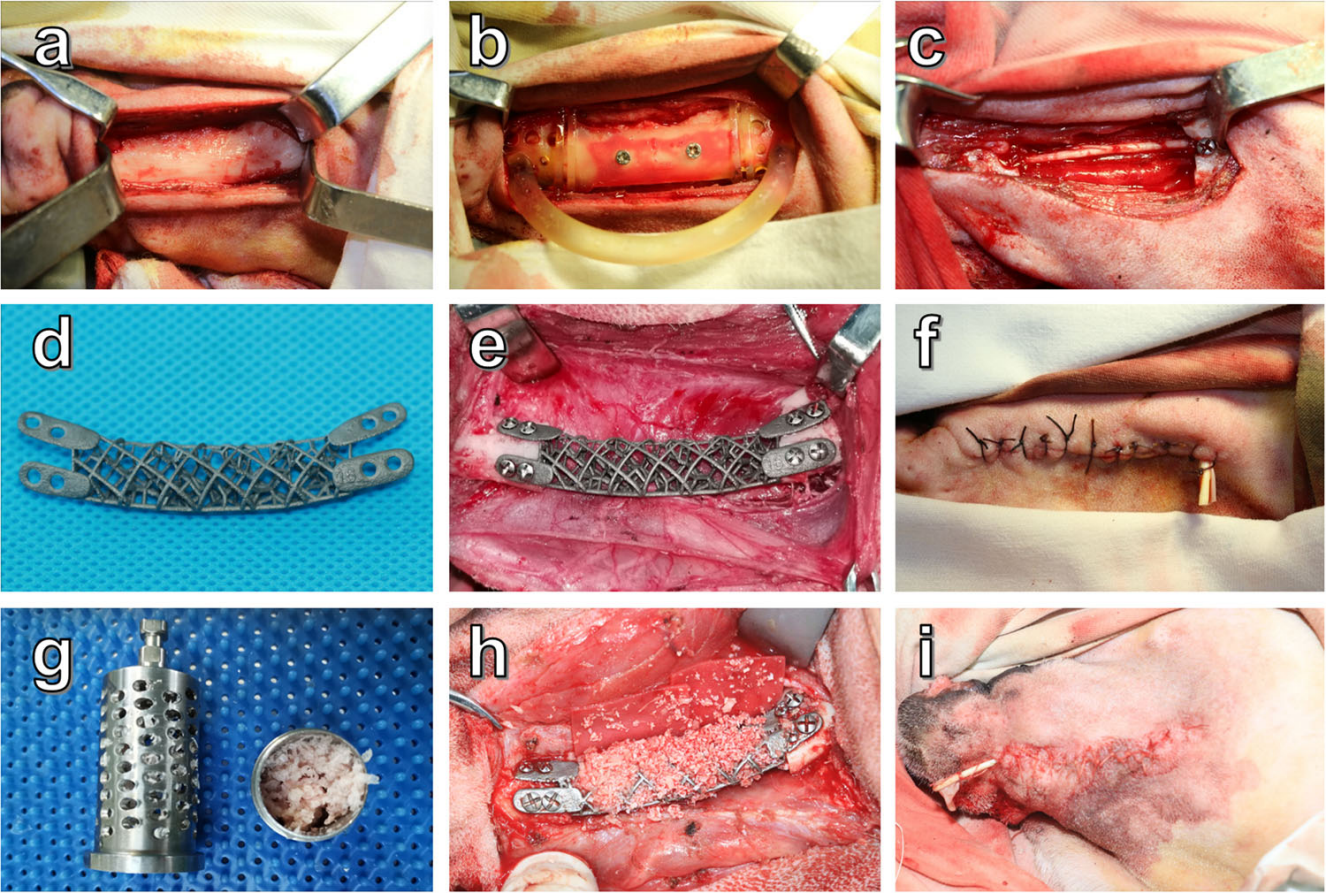
The procedure of scaffold implantation in animal experiments. **a** Exposure of the mandibular region. **b** Fixation of the guide plate. **c** 4 cm segment mandible defect preparation and inferior alveolar neurovascular bundle anatomy. **d** Ti-mesh scaffold. **e** Scaffold implantation and fixation. **f** Suture. **g** Autogenous bone fragment preparation by the bone mill. **h** Autogenous bone fragment combined with bone substitute filled into the pore of the scaffold. **i** Suture.

Twenty adult beagle dogs underwent a critically sized mandibular defect 4 cm in length, created on the side of the edentulate area. The procedure is illustrated in Fig. 7. The procedures of anesthesia, analgesia, and infection avoidance were according to previous teeth extraction surgery. After the incision from the extraoral skin, a 3D-printing guide plate was fixed at the buccal region of the mandible, and a 4-cm segmental defect was created using orthopedic micropower systems. Simultaneously, the inferior alveolar neurovascular bundle was anatomized for protection and located on the lingual side. Subsequently, the 3D-printing Ti grid scaffold was fixed with screws. Ten animals in Group 1 were treated with scaffold implantation alone, without grafting additional materials. For the ten animals in Group 2, the scaffold gaps were filled with autologous bone chips combined with bone substitutes (Bio-Oss, Geistlich Pharma AG, Switzerland) and covered with absorbable collagen (Bioguide, Geistlich Pharma AG, Wolhusen, Switzerland). After drainage strip placement, the subcutaneous tissues and skin were sutured layer-by-layer with interrupted sutures. Semi-fluid was fed to avoid excessive occlusal forces. The animals were then subjected to spiral CT under general anesthesia 18 months after surgery. However, the spiral CT scanning follow-up was incomplete due to the COVID-19 pandemic. One animal in each group was selected for the evaluation of bone metabolism in the scaffold, monitored by single-photon ECT/CT (SPECT/CT) 18 months after implantation.

After 18 months, all animals were euthanized according to the following procedures: the animals were monitored with an electrocardiogram before euthanasia, over anesthesia with pentobarbital (3%, 100 mg/kg) intravenous injection was applied before harvest, after vital signs including breath, heart rate was not detected, and tongue cyanosis observed, mandibles with scaffolds were harvest for micro-CT scanning and histological analysis. Calcein (8 mg kg^−1^. Sigma Chemicals Co., USA) and tetracycline (50 mg kg^−1^. Amresco Ltd., USA) was administered by intramuscular injection on days 4 and 14 before euthanized, and the animals were sacrificed to determine new bone regeneration and deposition.

### Micro-CT scanning

Before histological analysis, the two groups of samples were scanned using micro-CT (Siemens Inveon MM Micro CT, Germany). A 3D image was reconstructed with an isotropic voxel size of 80 µm. The multilevel threshold procedure (the threshold for bone and materials) was applied to discriminate bone from other tissues. Images acquired from the scans were used for quantitative analysis. Regions of interest (ROIs) comprised tissues within the scaffold. BV/TV were determined for analysis.

### Histological analysis

Samples were immersed in 75% alcohol for seven days and then dehydrated using a graded series of dilutions of ethanol and 100% acetone. Samples were embedded in polyester resin and mounted, and 100 µm-thick undecalcified sections were cut using a sawing microtome (Leica SP 1600, Leica Microsystems, Germany), with images captured using a fluorescence microscope (OLYMPUS BX53, Tokyo, Japan), and analyzed with the digitized image-analysis system (OsteoMeasure^TM^). Mineralized bone tissues were labeled using two different fluorescent markers. The distance between the two fluorescent markers (yellow and green) indicated the new bone mineralization ratio, defined as the mineral apposition ratio (MAR, µm/day).

After fluorescence observation-based evaluation, the sections were stained with methylene blue/acid fuchsin and analyzed using a digitized image-analysis system (OsteoMeasure^TM^) coupled with a light microscope (OLYMPUS BX53, Tokyo, Japan). The ROIs were determined as the profiles of the scaffolds corresponding to the osteotomy defect size, and the mineralized bone tissue and Ti-grid scaffolds were calculated as the percentage of the total area of the ROI.

### Statistical analysis

Data were analyzed using SPSS for Windows (version 26.0; IBM, Armonk, NY). As the normal distribution of data could not be confirmed owing to the small sample size, the data were expressed as median (interquartile range). The comparison of Group 1 and Group 2 was performed using the Mann-Whitney U test, while the comparison among the distal, middle, and mesial areas was performed using the Friedman test. Differences were considered statistically significant at *p* < 0.05.

### Clinical trials

Three patients were diagnosed with mandibular neoplasm in the Department of Oral and Maxillofacial Surgery, General Hospital of the Chinese People’s Liberation Army (PLA), and needed bone defect reconstruction after surgery. Each patient received an individual Ti-grid scaffold design and manufactured using a metal 3D-printing technique (Supplementary Fig. 7, Supplementary Fig. 11, and Fig. 6). The Ti-grid scaffold corresponded to the range of the osteotomized mandible. The details of the three clinical trials are as follows:

Case 1: A 3-year-old female patient experienced feeding and breathing difficulties due to compression from a large neoplasm of the mandibular body. Preoperative pathological examination revealed a cementoma. Spiral CT images indicated that the range of the neoplasm was located at the front edge of the bilateral mandibular ramus. The range of the segmental defects was approximately 10 cm (Supplementary Fig. 6). Considering the patient’s age and the characteristics of the neoplasm, the patient would need numerous operations for mandibular reconstruction, autogenous bone transplantation from the iliac crest, ribs, or fibula bone, (which were relatively small), did not meet the requirements of reconstruction and secondary bone graft surgery. A reconstructed plate (r-plate) may result in a fracture or exposure owing to bending in advance. Therefore, a reconstruction plan fabricated with Ti grid scaffold implantation was proposed in our study (Fig. 5). The scaffold was fabricated using SLM. To ensure accuracy, neoplasm resection and scaffold implantation were performed using a guide plate. Autologous iliac bone fragments were prepared using a bone mill (USTOMED, Germany), and the inferior alveolar neurovascular bundles were reserved and placed on the lingual side of the mandible during the surgery (Fig. 5). The results from postoperative paraffin sectioning indicated myxofibroma. Spiral CT was performed at 3, 5, 16, 24, and 36 months postoperatively. The ECT was applied to evaluate bone metabolism in the scaffold 36 months after surgery.

Case 2: A 39-year-old male presented with a painless neoplasm in the left facial region accompanied by trismus for approximately five months (Supplementary Fig. 9). Spiral CT images indicated apparent bone destruction of the mandibular body and ramus (Supplementary Fig. 14). Based on bone destruction, we designed a Ti grid scaffold ranging from the distal portion of the left lower second premolar to the temporomandibular joint (Supplementary Fig. 10). The range of segmental defects was approximately 7 cm. Considering the long-term occlusal impact at this age, R-plate restoration was excluded because of the risk of fracture and exposure. At the same time, the patient refused autogenous bone transplantation for hard physical labor. Therefore, a 3D printing mandibular scaffold was fabricated for reconstruction, and the temporomandibular fossa was manufactured with high polymer polyethylene via a subtractive manufacturing technique (Supplementary Fig. 11). The neoplasm was resected under operative guidance. Subsequently, an individualized, patient-specific Ti grid scaffold was implanted without any other material filled (Supplementary Fig. 12). To avoid occlusion disorders, the scaffold placement was completed based on intermaxillary ligation. The results from the intraoperative fast-frozen and postoperative paraffin sections indicated squamous cell carcinoma. Spiral CT was performed 3 and 6 months after surgery.

Case 3: A 64-year-old male patient underwent titanium plate fracture reconstruction. The patient underwent a right mandible segmental section due to squamous cell carcinoma and Ti plate reconstruction 1 year before the Ti plate fracture. Local head-neck radiotherapy was performed 1 month after the surgery. No signs of tumor recurrence were observed after postoperative follow-up. No exposure of the skin or intraoral gingiva was observed. The range of the segmental defects was approximately 4 cm. Treatment with an r-plate was excluded because of the risk of fracture again. Autogenous bone transplantation was excluded for the poor vascular condition at the recipient site, which resulted from previous surgery and radiotherapy. A mechanically optimized patient-specific Ti-grid scaffold (Fig. 6) was planned for the patient. Implantation of the scaffold was performed via a previous submandibular approach. After the scaffold was fixed on the defect area, autologous iliac bone fragments filled the pores of the scaffold (Fig. 6). Finally, an extraoral incision was sutured in layers.

### Reporting summary

Further information on research design is available in the Nature Research Reporting Summary linked to this article.

## Supplementary information

Supplemental Material

Reporting Summary

## Data Availability

All data needed to evaluate or reproduce the conclusions are available in the paper and/or the Supplementary materials. All datasets generated during and/or analyzed during the current study are available from the corresponding author upon reasonable request when qualified researchers contact, and a data usage agreement signing was necessary.

## Code availability

No custom code or mathematical algorithm was used in this study. Experimental data statistics in this study were performed using SPSS v26.0 and obtained and presented as expressed as median (interquartile range). The comparison of Group 1 and Group 2 was performed using the Mann–Whitney *U* test, while the comparison among the distal, middle, and mesial areas was performed using the Friedman test. Differences were considered statistically significant at *p* < 0.05.
