# Supplementary material for: 3D printing titanium grid scaffold facilitates osteogenesis in mandibular segmental defects
Source: NPJ Regen Med. 2023 Jul 24;8:38. doi: 10.1038/s41536-023-00308-0 (PMC10366137; doi:10.1038/s41536-023-00308-0)
Supplement: Supplementary file 1 — Supplemental Material [file 41536_2023_308_MOESM1_ESM.pdf]

3D printing titanium grid scaffold facilitates osteogenesis in mandibular segmental defects

Yongfeng Li[1], Huawei Liu[1], ChaoWang[2], Rongzeng Yan[3], Lei Xiang[1], Xiaodan Mu[1], Lingling Zheng[2], Changkui Liu[4], Min Hu[1,*]

[1] Department of Stomatology, the First Medical Center of PLA General Hospital, Beijing China

[2] Beijing Advanced Innovation Center for Biomedical Engineering, Beihang University, Beijing 100083, China

[3] Nanchang University Fuzhou Medical College, Fuzhou 344000, China

[4]Department of Oral and Maxillofacial Surgery, School of Stomatology, Xi'an Medical University, Xi'an, China.

* Corresponding Author. Email: humin48@vip.163.com (M.H.)

Table of contents

Supplementary Figure 1. Ti-mesh scaffold design and fabrication for animal experiment.

Supplementary Figure 2. Spiral CT scanning of the experimental animals 18 months after scaffold implantation.

Supplementary Figure 3. SPECT/CT scanning of the experimental animals 18 months after scaffold implantation.

Supplementary Figure 4. Micro-CT scanning after experimental animals sacrificed 18 months after scaffold implantation.

Supplementary Figure 5. Double-fluorescence labeling observation of the new bone mineralization:

Supplementary Figure 6. Scaffold design of case 1.

Supplementary Figure 7. Scaffold fabrication of case 1.

Supplementary Figure 8. Bone metabolism evaluation from ECT scanning of case 1.

Supplementary Figure 9. Clinical observation of case 2 before operation.

Supplementary Figure 10. Scaffold design of case 2.

Supplementary Figure 11. Scaffold fabrication of case 2.

Supplementary Figure 12. The procedures of scaffold implantation in case 2.

Supplementary Figure 13. Clinical observation of case 2 three months after operation.

Supplementary Figure 14. Spiral CT scanning following up of case 2.

Supplementary Figure 15. Clinical observation of case 3 one month after operation.

Supplementary Figure 16. Spiral CT scanning following up of case 3.

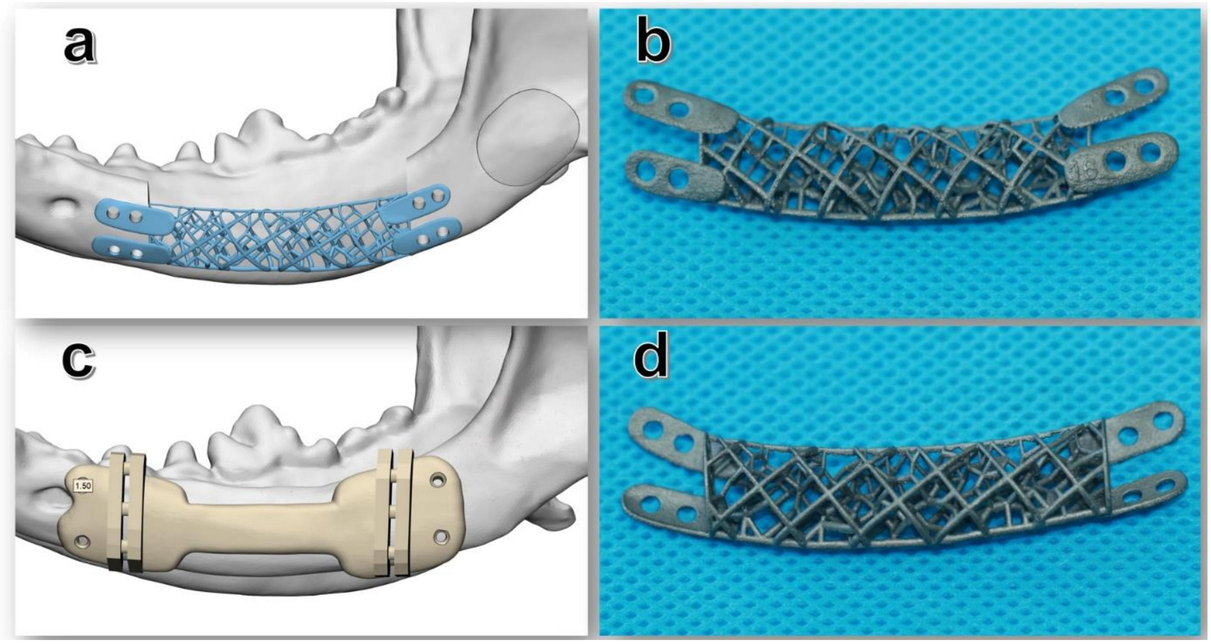

Supplementary Figure 1. Ti-mesh scaffold design and fabrication for animal experiment:

a. Design of scaffold;

b. Buccal view of the scaffold;

c. Design of guide plate;

d. Lingual view of the scaffold.

‘

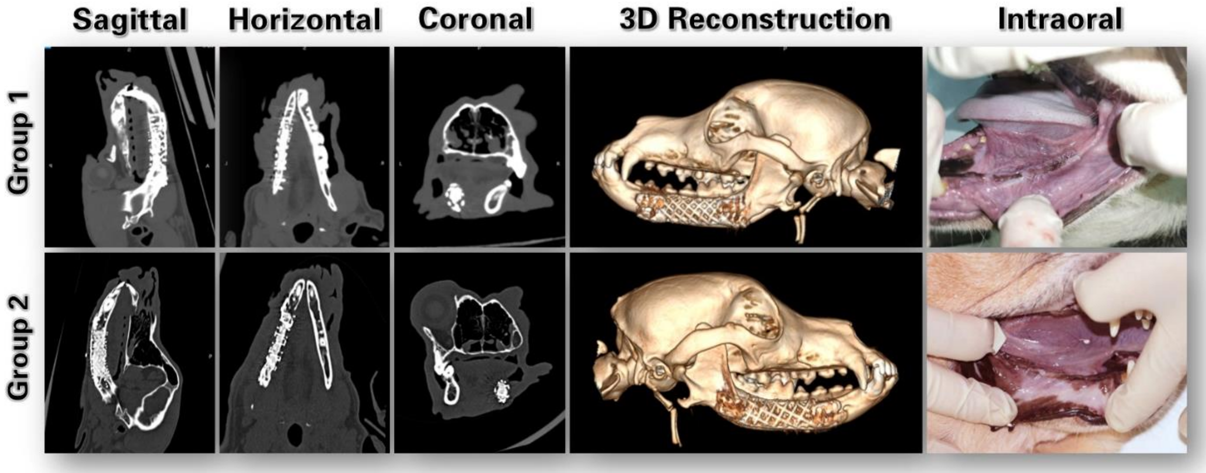

Supplementary Figure 2. Spiral CT scanning of the experimental animals 18 months after scaffold implantation.

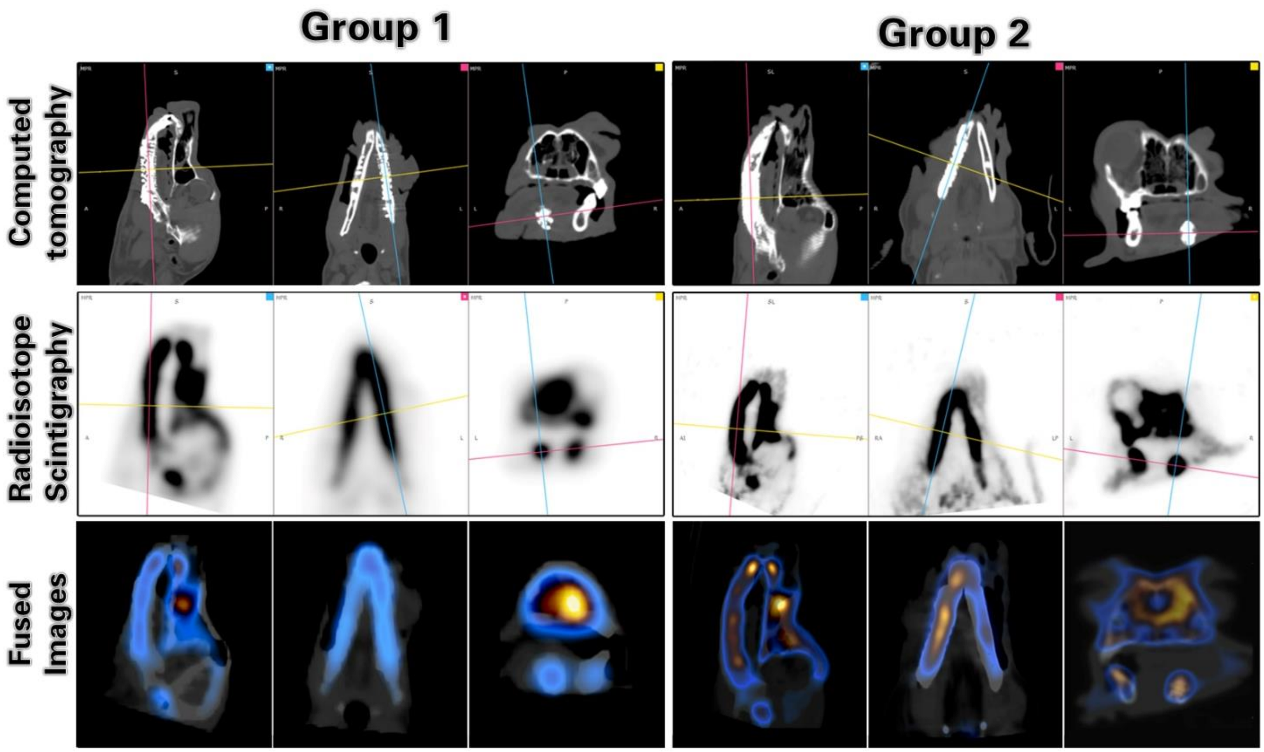

Supplementary Figure 3. SPECT/CT scanning of the experimental animals 18 months after scaffold implantation.

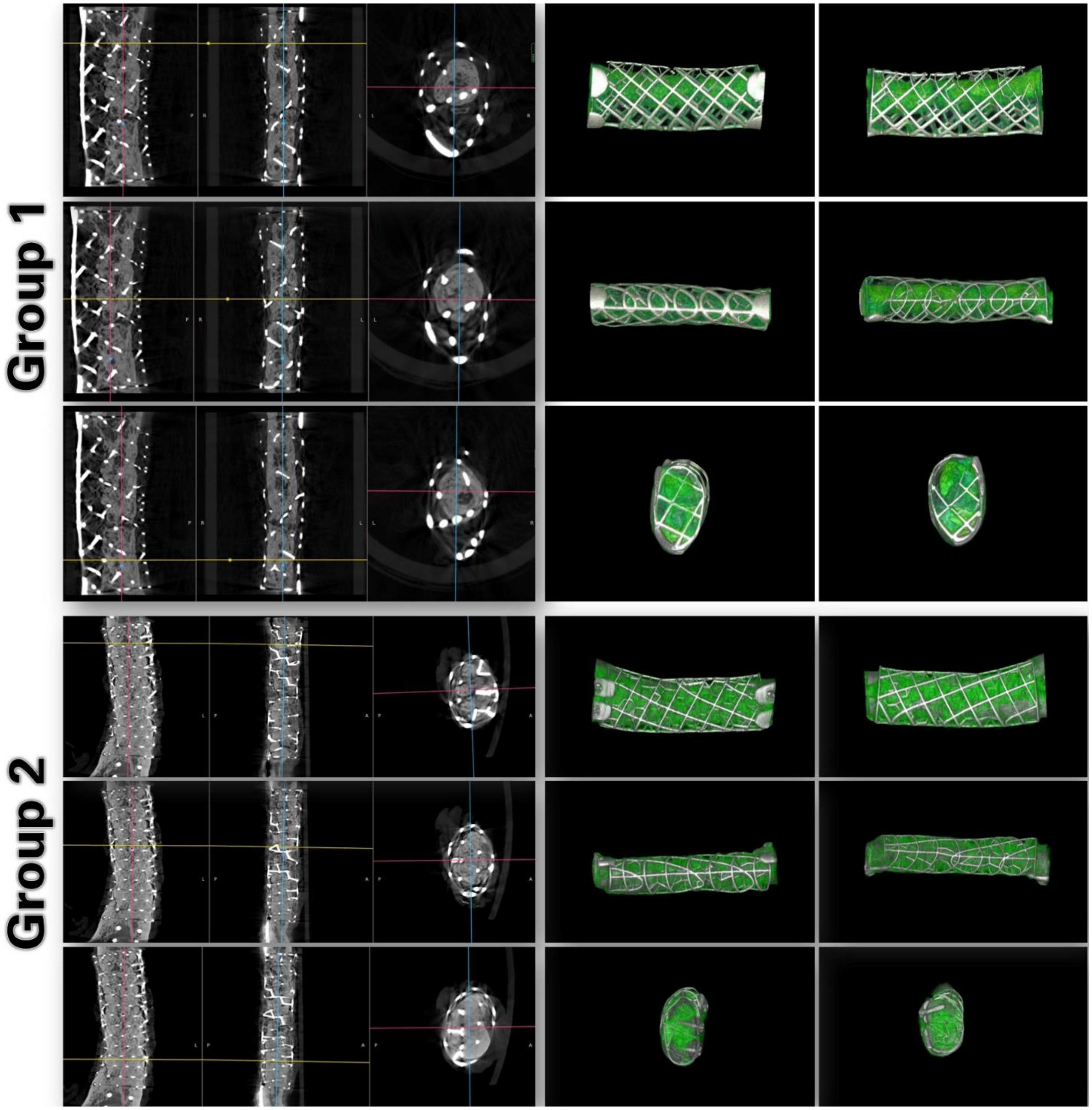

Supplementary Figure 4. Micro-CT scanning after experimental animals sacrificed 18 months after scaffold implantation.

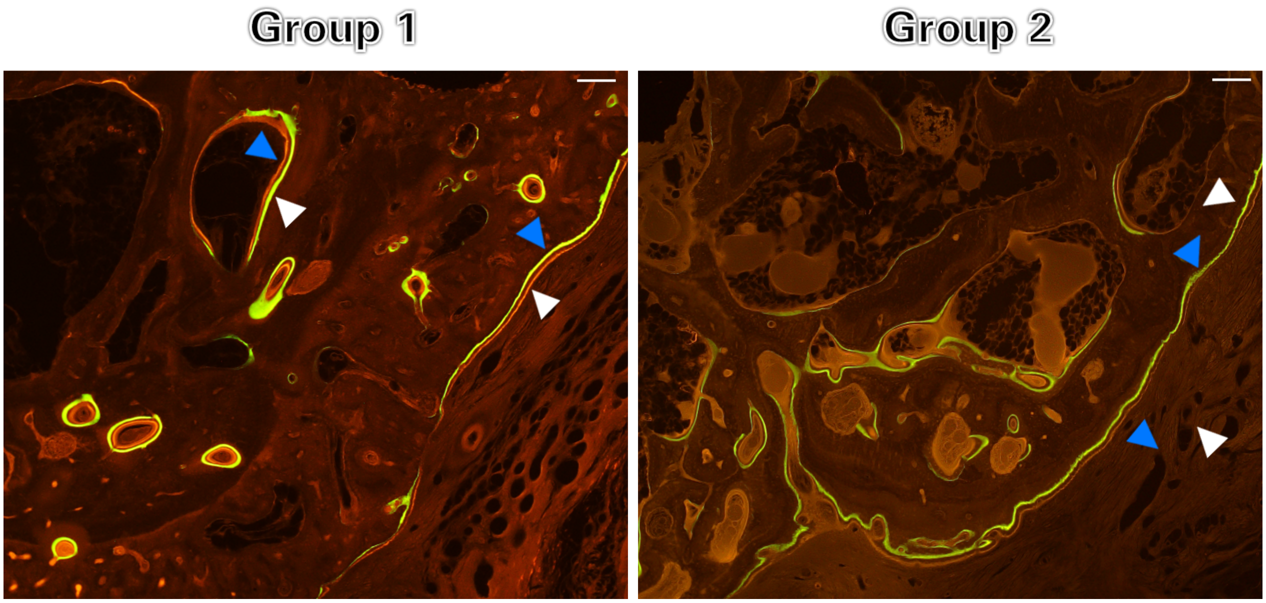

Supplementary Figure 5. Double-fluorescence labeling observation of the new bone mineralization: Tetracycline were observed with a yellow fluorescence marker (blue triangles); Calcein were observed with a green fluorescence marker (white triangles). Scale bar, 200μm.

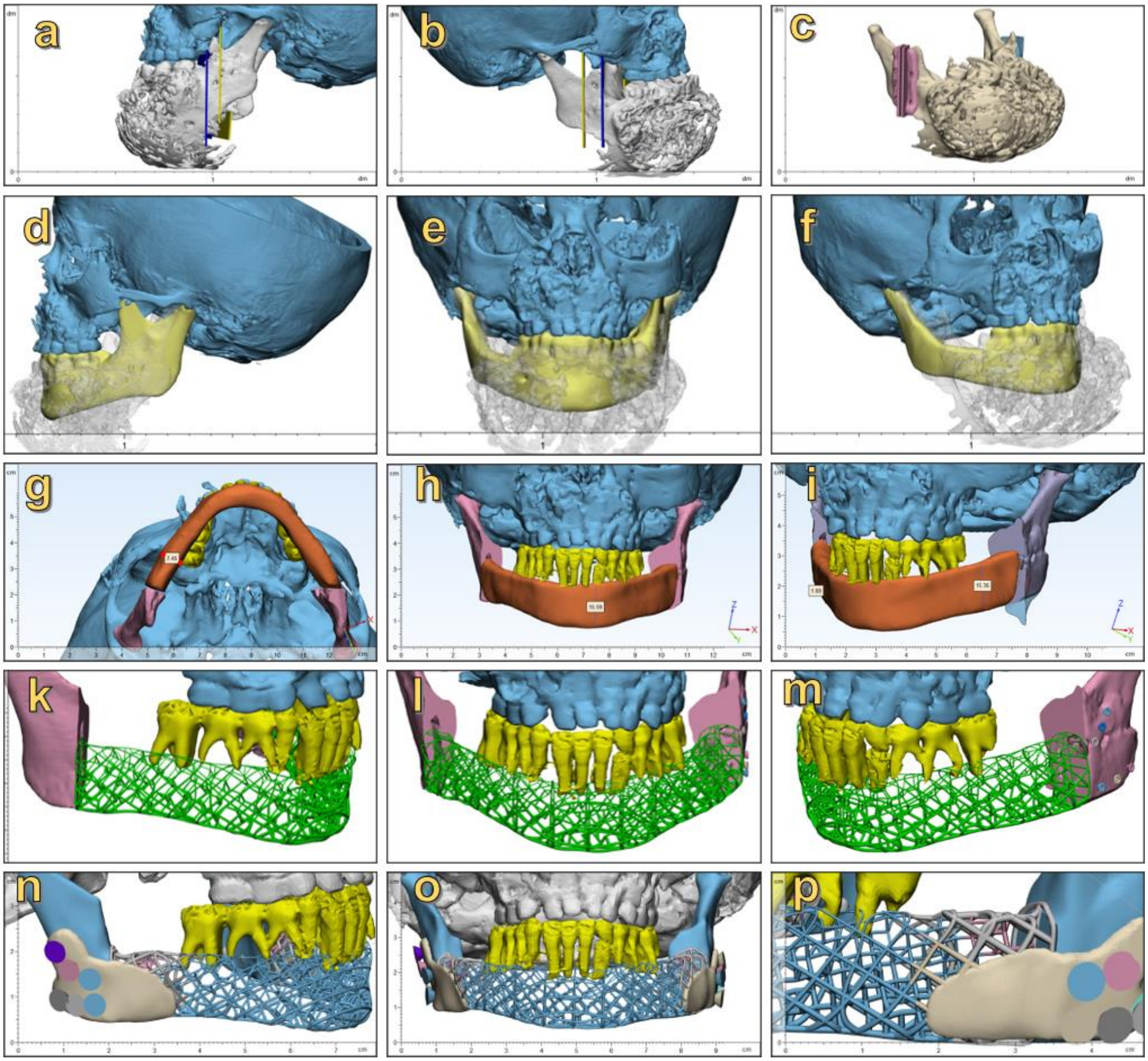

Supplementary Figure 6. Scaffold design of case 1:

a-c. Osteotomy area confirmation;

d-e. Simulation of prospected mandible profile;

g-i. Scaffold profile; k-m. Mesh structure of the scaffold;

n-p. The Ti-mesh scaffold structure and fixation region.

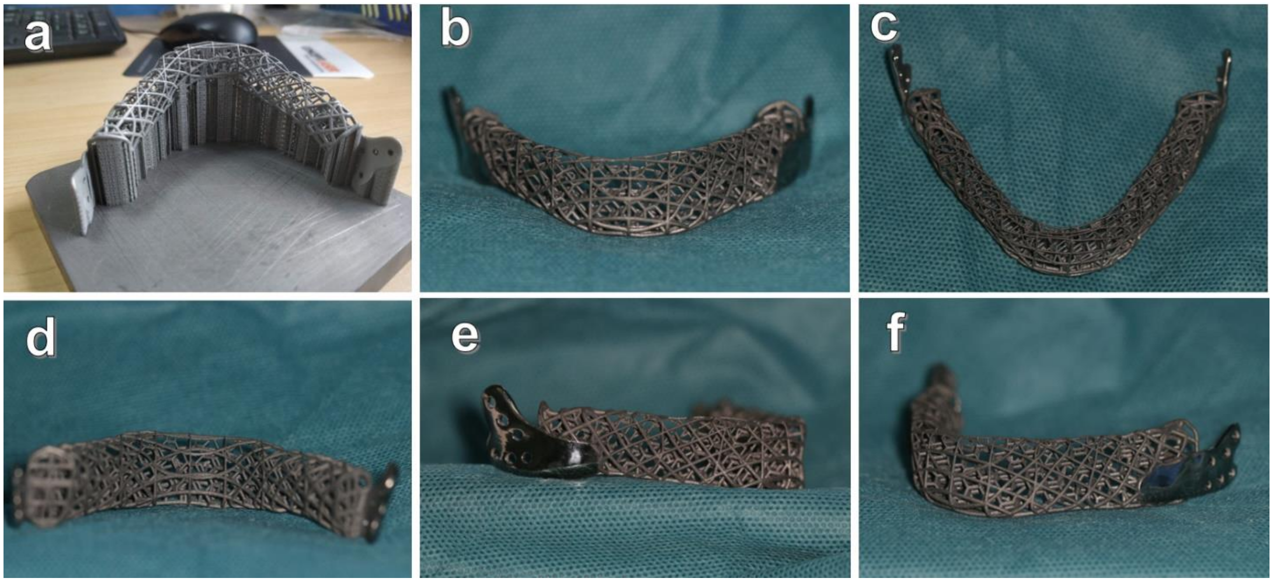

Supplementary Figure 7. Scaffold fabrication of case 1:

a. Scaffold before support part remove; b. Anterior view of the scaffold;

c. Superior view of the scaffold; d. Posterior view of the scaffold;

e. Right view of the scaffold; f. Left view of the scaffold.

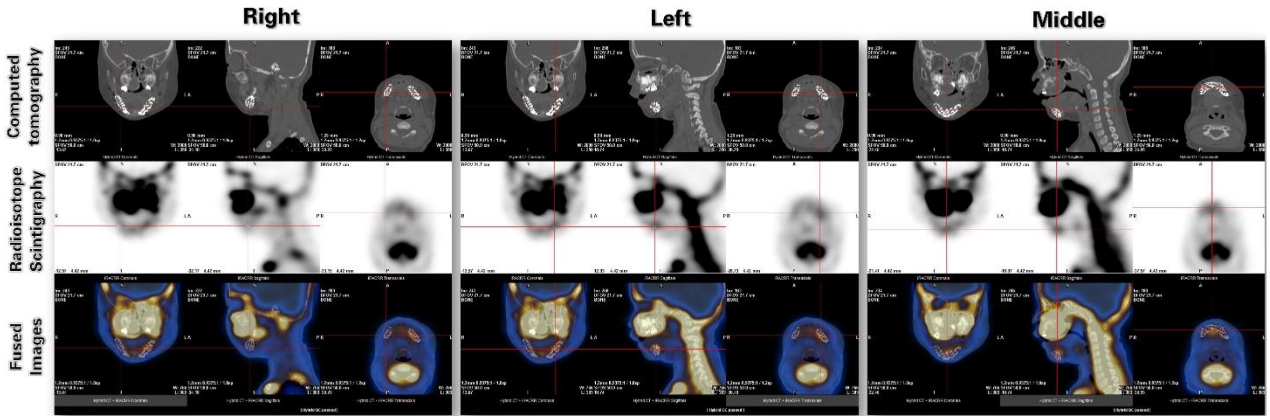

Supplementary Figure 8. Bone metabolism evaluation from ECT scanning of case 1.

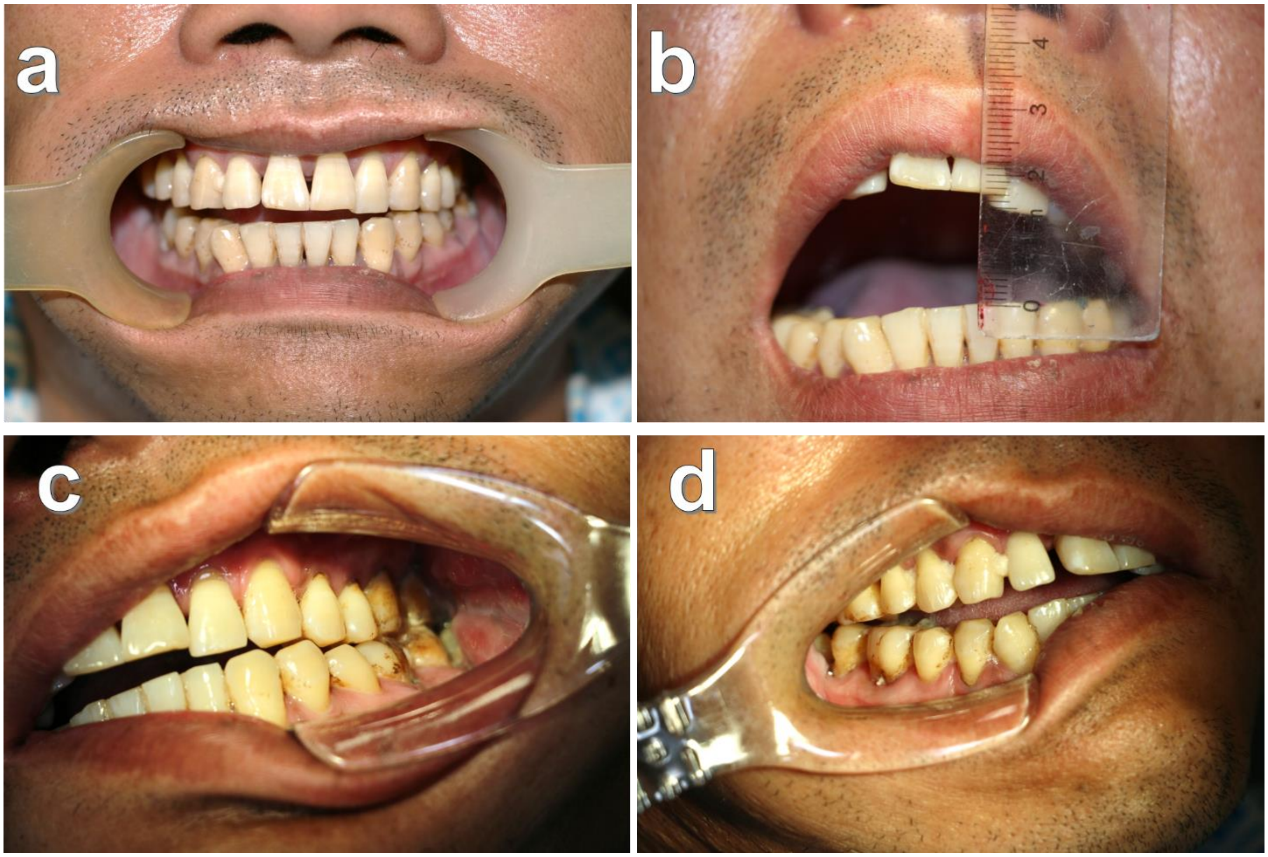

Supplementary Figure 9. Clinical observation of case 2 before operation.

a. Anterior view of mouth closing position;

b. Anterior view of mouth opening position;

c. Left view of mouth closing position;

d. Right view of mouth closing position.

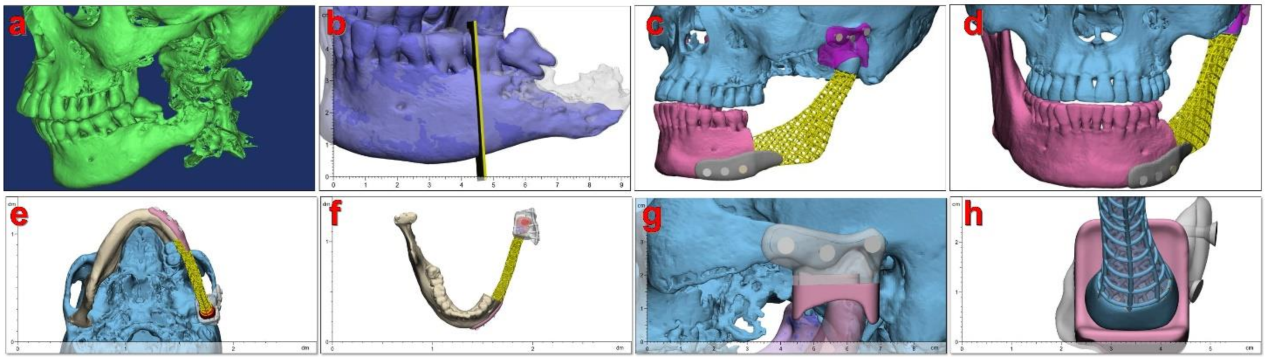

Supplementary Figure 10. Scaffold design of case 2:

a. 3D reconstruction images of case 2 before operation;

b. Osteotomy area confirmation; c-f. Mesh structure of the scaffold;

g-h. Design of temporal-mandibular joint.

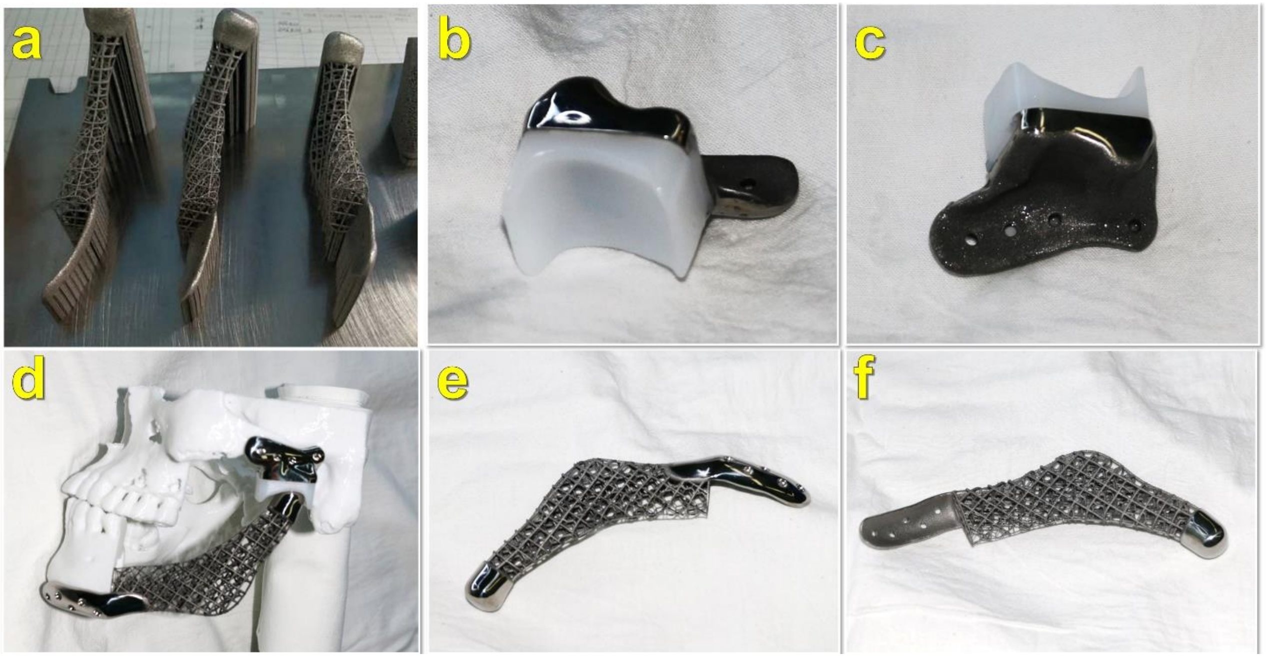

Supplementary Figure 11. Scaffold fabrication of case 2:

a. Scaffold before support part remove;

b. Inferior view of the temporomandibular fossa prosthesis;

c. Superior view of the temporomandibular fossa prosthesis;

d. Assembling of Ti-mesh scaffold and the temporomandibular fossa prosthesis;

e. Buccal view of the Ti-mesh scaffold;

f. Inferior view of the scaffold.

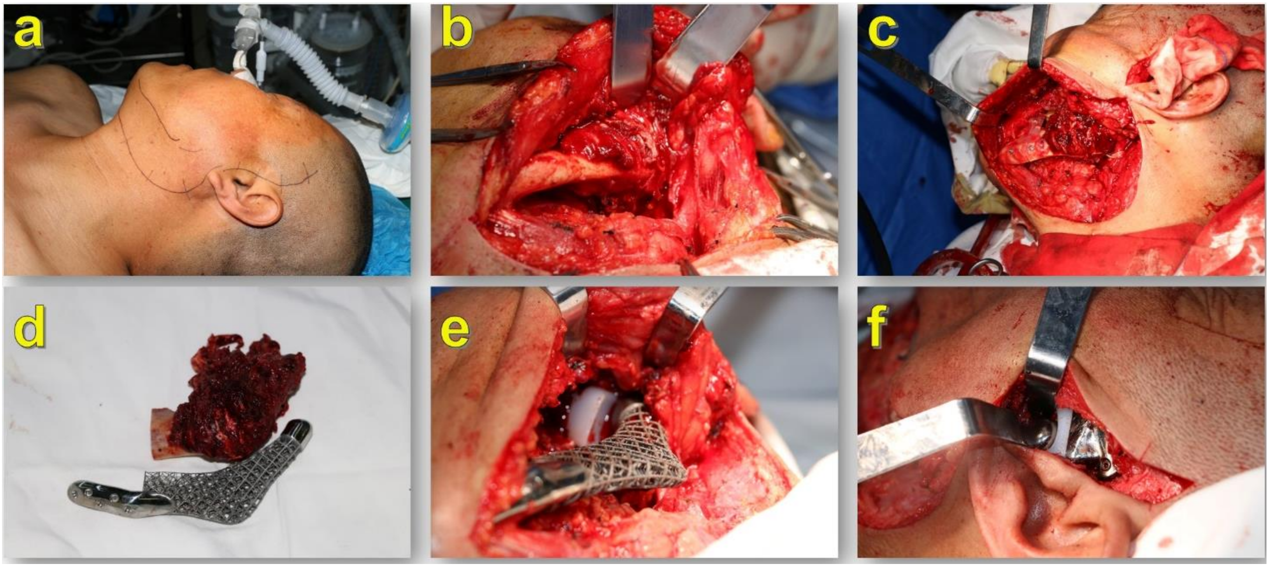

Supplementary Figure 12. The procedures of scaffold implantation in case 2:

a. Surgical incision design;

b. Exposure of neoplasm;

c. Guide plate fixation;

d. Neoplasm excision;

e. Scaffold implantation and fixation;

f. Temporomandibular fossa prosthesis fixation.

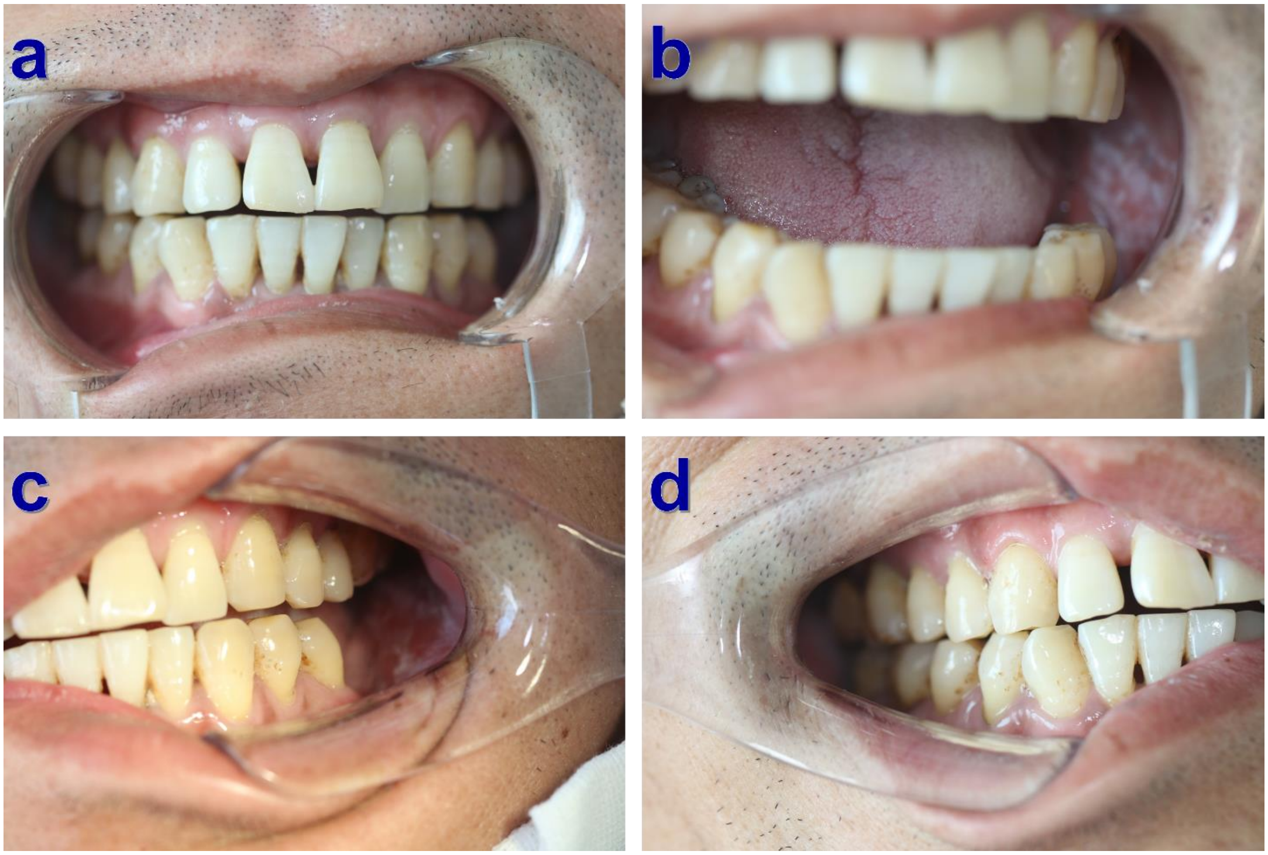

Supplementary Figure 13. Clinical observation of case 2 three months after operation.

a. Anterior view of mouth closing position;

b. Anterior view of mouth opening position;

c. Left view of mouth closing position;

d. Right view of mouth closing position.

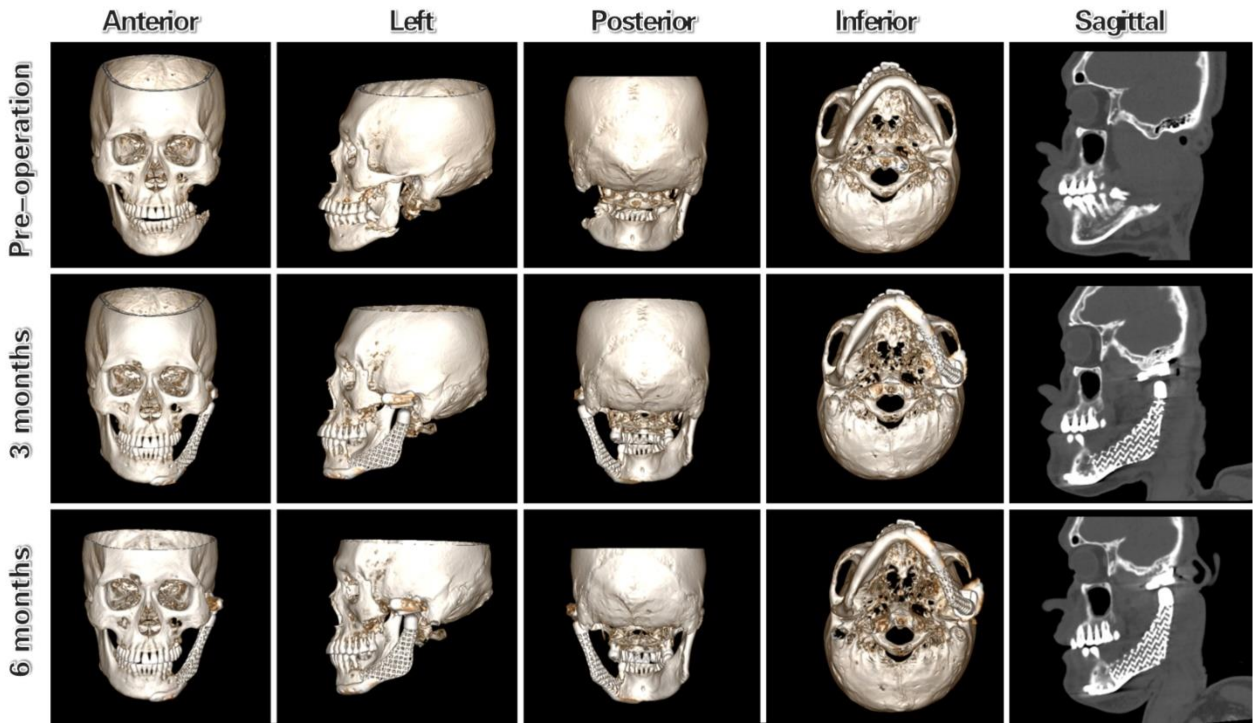

Supplementary Figure 14. Spiral CT scanning following up of case 2.

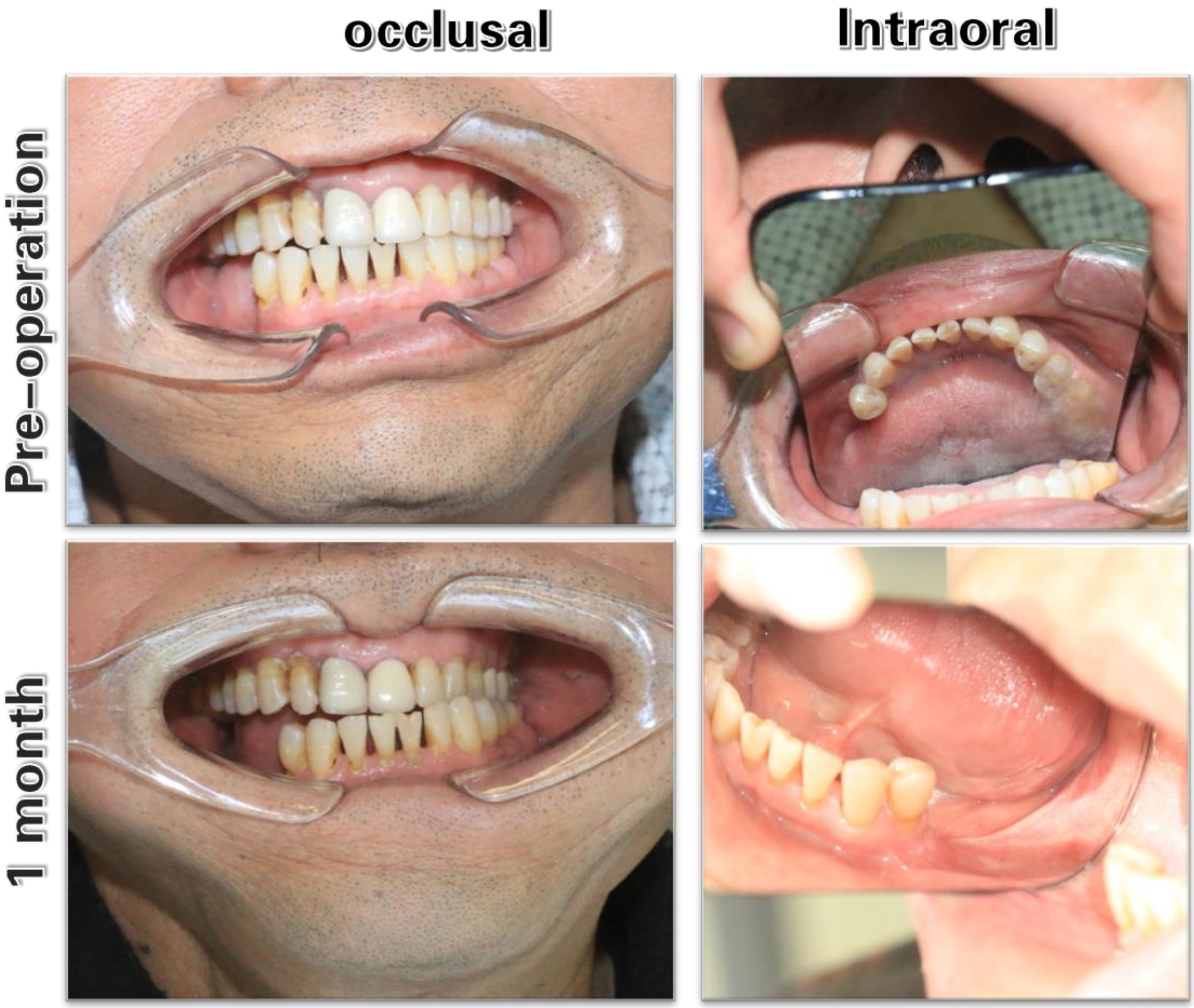

Supplementary Figure 15. Clinical observation of case 3 one month after operation.

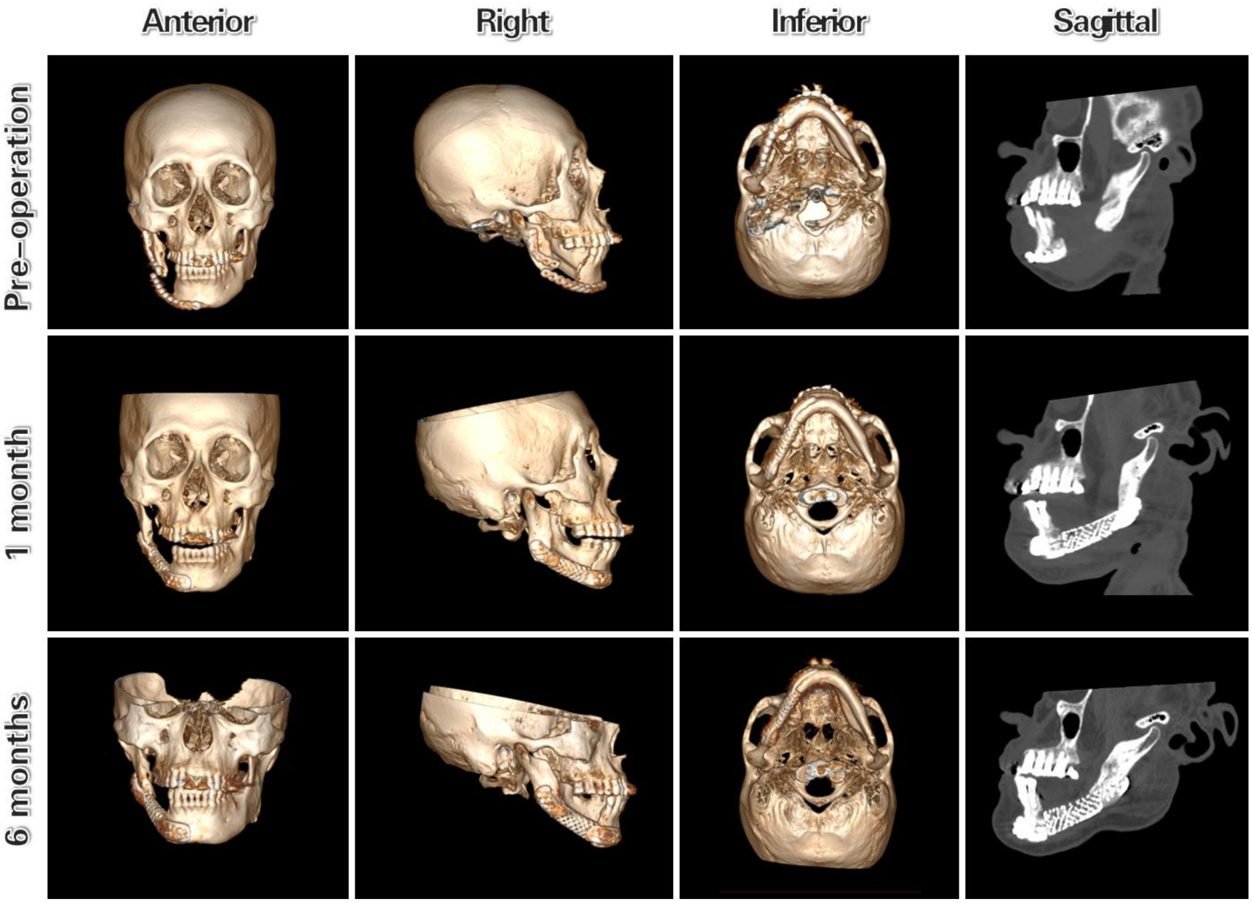

Supplementary Figure 15. Spiral CT scanning following up of case 3.
