# Supplementary material for: 3D printing titanium grid scaffold facilitates osteogenesis in mandibular segmental defects
Source: NPJ Regen Med. 2023 Jul 24;8:38. doi: 10.1038/s41536-023-00308-0 (PMC10366137; doi:10.1038/s41536-023-00308-0)
Supplement: Supplementary file 2 — Reporting Summary [file 41536_2023_308_MOESM2_ESM.pdf]

nature portfolio

Corresponding author(s): Min Hu

Last updated by author(s): Dec 2, 2022

Reporting Summary

Nature Portfolio wishes to improve the reproducibility of the work that we publish. This form provides structure for consistency and transparency in reporting. For further information on Nature Portfolio policies, see our Editorial Policies and the Editorial Policy Checklist.

Statistics

For all statistical analyses, confirm that the following items are present in the figure legend, table legend, main text, or Methods section.

n/a	Confirmed	
☐	☒	The exact sample size (n) for each experimental group/condition, given as a discrete number and unit of measurement
☒	☐	A statement on whether measurements were taken from distinct samples or whether the same sample was measured repeatedly
☐	☒	The statistical test(s) used AND whether they are one- or two-sided<br>*Only common tests should be described solely by name; describe more complex techniques in the Methods section.*
☒	☐	A description of all covariates tested
☐	☒	A description of any assumptions or corrections, such as tests of normality and adjustment for multiple comparisons
☐	☒	A full description of the statistical parameters including central tendency (e.g. means) or other basic estimates (e.g. regression coefficient) AND variation (e.g. standard deviation) or associated estimates of uncertainty (e.g. confidence intervals)
☐	☒	For null hypothesis testing, the test statistic (e.g. F, t, r) with confidence intervals, effect sizes, degrees of freedom and P value noted<br>*Give P values as exact values whenever suitable.*
☒	☐	For Bayesian analysis, information on the choice of priors and Markov chain Monte Carlo settings
☒	☐	For hierarchical and complex designs, identification of the appropriate level for tests and full reporting of outcomes
☒	☐	Estimates of effect sizes (e.g. Cohen's d, Pearson's r), indicating how they were calculated

Our web collection on statistics for biologists contains articles on many of the points above.

Software and code

Policy information about availability of computer code

Data collection	There was no software used for data collection in this study.
Data analysis	In this study, the grid structures were developed using 3-Matic software 12.0 (Materialize, Leuven, Belgium) and the data were analyzed using SPSS for Windows (version 26.0; IBM, Armonk, NY).

For manuscripts utilizing custom algorithms or software that are central to the research but not yet described in published literature, software must be made available to editors and reviewers. We strongly encourage code deposition in a community repository (e.g. GitHub). See the Nature Portfolio guidelines for submitting code & software for further information.

Data

Policy information about availability of data

All manuscripts must include a data availability statement. This statement should provide the following information, where applicable:

- Accession codes, unique identifiers, or web links for publicly available datasets
- A description of any restrictions on data availability
- For clinical datasets or third party data, please ensure that the statement adheres to our policy

The data that support the findings of this study are available from the corresponding author upon reasonable request.

Human research participants

Policy information about studies involving human research participants and Sex and Gender in Research.

Reporting on sex and gender	Three patients involved in this study, consisted of two males and one female, the sex and gender was not considered as determining factors in this study.
Population characteristics	Three patients involved in this study, the details of the three clinical trials are as follows: Case 1: A 3-year-old female patient; Case 2: A 39-year-old male; Case 3: A 64-year-old female patient.
Recruitment	Three patients were diagnosed with mandibular neoplasm in the Department of Oral and Maxillofacial Surgery, General Hospital of the Chinese People's Liberation Army (PLA), and needed bone defect reconstruction after surgery.
Ethics oversight	This study was approved and supervised by the Medical Ethics Committee of the Chinese PLA General Hospital on March 28th 2019 (Beijing, China. approval No. S2019-065-01).

Note that full information on the approval of the study protocol must also be provided in the manuscript.

Field-specific reporting

Please select the one below that is the best fit for your research. If you are not sure, read the appropriate sections before making your selection.

☒ Life sciences ☐ Behavioural & social sciences ☐ Ecological, evolutionary & environmental sciences

For a reference copy of the document with all sections, see nature.com/documents/nr-reporting-summary-flat.pdf

Life sciences study design

All studies must disclose on these points even when the disclosure is negative.

Sample size

Sample-size calculation was not performed in this study. The sample-size was determined by two similar studies published at recent years 1,2which applied larger animals for animal experiment. The two studies involved 5 animals/4 groups and 6 animals/4 groups, respectively. So we determined 10 animals in each group.

References

1 Kengelbach-Weigand, A. et al. Personalized medicine for reconstruction of critical-size bone defects - a translational approach with customizable vascularized bone tissue. NPJ Regen Med 6, 49 (2021). https://doi.org:10.1038/s41536-021-00158-8.
2 Pobloth, A. M. et al. Mechanobiologically optimized 3D titanium-mesh scaffolds enhance bone regeneration in critical segmental defects in sheep. Sci Transl Med 10 (2018). https://doi.org:10.1126/scitranslmed.aam8828.

Data exclusions	Three beagle dogs (including one in Group 2 and two in Group 1) were excluded from this study because of anesthesia or scaffold fracture due to unanticipated overloaded biting (i.e., metal cage).
Replication	The design and fabrication of the scaffold were accomplished by the same design group and the similar 3D printing technique and post-treatment processing. At the same time, the scaffolds implantation surgery of animal experiment and clinical trials were carried out by the same operators and with the guidance of guide plate. The radiological scanning and histological analysis were performed at the same institution .
Randomization	The animal were random allocated to Group 1 and Group 2 with Random number table method.
Blinding	The investigators were blinded to group allocation during data collection and analysis in animal experiment, the surgical side and grouping were random allocation.

Reporting for specific materials, systems and methods

We require information from authors about some types of materials, experimental systems and methods used in many studies. Here, indicate whether each material, system or method listed is relevant to your study. If you are not sure if a list item applies to your research, read the appropriate section before selecting a response.

Materials & experimental systems

n/a	Involved in the study
☒	☐ Antibodies
☒	☐ Eukaryotic cell lines
☒	☐ Palaeontology and archaeology
☐	☒ Animals and other organisms
☐	☒ Clinical data
☒	☐ Dual use research of concern

Methods

n/a	Involved in the study
☒	☐ ChIP-seq
☒	☐ Flow cytometry
☒	☐ MRI-based neuroimaging

Animals and other research organisms

Policy information about studies involving animals; ARRIVE guidelines recommended for reporting animal research, and Sex and Gender in Research

Laboratory animals	Twenty adult beagle dogs (weight: 12 ± 3.4 kg; age: >2 years) were used in this study.
Wild animals	The study did not involve wild animals.
Reporting on sex	In this study,the findings were not applied to only one sex, sex was not considered in this study design.
Field-collected samples	The study did not involve samples collected from field. All animals were euthanized and mandibles with scaffolds were harvested for the subsequent test.
Ethics oversight	This study was approved and supervised by the Institutional Animal Care and Use Committee of the Chinese PLA General Hospital (Beijing, China. approval no. 2017-D13-15).

Note that full information on the approval of the study protocol must also be provided in the manuscript.

Clinical data

Policy information about clinical studies

All manuscripts should comply with the ICMJE guidelines for publication of clinical research and a completed CONSORT checklist must be included with all submissions.

Clinical trial registration	This study was approved and supervised by the Medical Ethics Committee of the Chinese PLA General Hospital on March 28th 2019 (Beijing, China. approval No. S2019-065-01).
Study protocol	The clinical tries in this study strictly complied with the requirements of Declaration of Helsinki, and was approved and supervised by the Medical Ethics of the Chinese PLA General Hospital on March 28th 2019 (Beijing, China. approval document No. S2019-065-01). Informed consent was obtained from patients or guardians of patients. The study was conducted in strict accordance with the requirements of the Medical Ethics Committee throughout the clinical trial.
Data collection	Three patients involved in this study, the data collection were completed in Chinese PLA General Hospital, the following-up ranged from 6 months to 3 years after surgery.
Outcomes	The facial appearance and radiological examination to assess the clinical outcomes in clinical trials, we defined no scaffolds fracture and mineralized new bone formed as fine outcomes.
